# Metabolic memory: mechanisms and diseases

**DOI:** 10.1038/s41392-024-01755-x

**Published:** 2024-02-28

**Authors:** Hao Dong, Yuezhang Sun, Lulingxiao Nie, Aimin Cui, Pengfei Zhao, Wai Keung Leung, Qi Wang

**Affiliations:** 1 State Key Laboratory of Oral Diseases & National Center for Stomatology & National Clinical Research Center for Oral Diseases, West China Hospital of Stomatology, Sichuan University, Chengdu, China; 2 Department of Prosthodontics, West China Hospital of Stomatology, Sichuan University, Chengdu, China; 3 Periodontology and Implant Dentistry Division, Faculty of Dentistry, The University of Hong Kong, Hong Kong, China

**Keywords:** Endocrine system and metabolic diseases, Molecular medicine, Metabolic disorders, Cancer metabolism

## Abstract

Metabolic diseases and their complications impose health and economic burdens worldwide. Evidence from past experimental studies and clinical trials suggests our body may have the ability to remember the past metabolic environment, such as hyperglycemia or hyperlipidemia, thus leading to chronic inflammatory disorders and other diseases even after the elimination of these metabolic environments. The long-term effects of that aberrant metabolism on the body have been summarized as metabolic memory and are found to assume a crucial role in states of health and disease. Multiple molecular mechanisms collectively participate in metabolic memory management, resulting in different cellular alterations as well as tissue and organ dysfunctions, culminating in disease progression and even affecting offspring. The elucidation and expansion of the concept of metabolic memory provides more comprehensive insight into pathogenic mechanisms underlying metabolic diseases and complications and promises to be a new target in disease detection and management. Here, we retrace the history of relevant research on metabolic memory and summarize its salient characteristics. We provide a detailed discussion of the mechanisms by which metabolic memory may be involved in disease development at molecular, cellular, and organ levels, with emphasis on the impact of epigenetic modulations. Finally, we present some of the pivotal findings arguing in favor of targeting metabolic memory to develop therapeutic strategies for metabolic diseases and provide the latest reflections on the consequences of metabolic memory as well as their implications for human health and diseases.

## Introduction

Metabolic diseases place a significant burden on global health systems.^1^ The prevalence of several metabolic diseases, encompassing diabetes, obesity as well as metabolism-associated fatty liver disease (MAFLD), has steadily increased over the past few decades. According to the Diabetes Atlas published in 2021, diabetes affects approximately 10.5% of adults globally, and the absolute amount of individuals suffering from diabetes is projected to rise in 2045 by 46%.^2^ Similarly, the prevalence of obesity and overweight exhibits a comparable growth pattern to that of diabetes.^3^ Since 1975, the prevalence of obesity increased almost twofold globally, with over 1.9 billion individuals categorized as overweight or obese by 2016.^4^ Approximately 25–30% of people worldwide are affected by MAFLD, which is the most common liver disease globally and has prevalence and incidence rates aligned with the escalating trends of obesity as well as type 2 diabetes mellitus (T2DM)^5,6^ Increasing incidences of these aberrant metabolism-related diseases and their consequential serious complications pose significant health challenges to human society. Consequently, it is imperative to investigate the pathological mechanisms involved in metabolic diseases as well as develop therapeutic interventions based on these scientific findings.

The initiation and evolution of metabolic diseases involve intricate mechanisms, requiring comprehensive therapeutic approaches for effective management.^7–9^ Recent studies have elucidated the persistent detrimental consequences that arise when cells are exposed to an abnormal metabolic environment. Even after the metabolic environment returns to a normal state, the cellular changes and characteristics of the abnormal metabolic state persist.^10–12^ These enduring cellular changes and characteristics represent the organism’s memory of an earlier metabolic state, exemplifying the phenomenon termed metabolic memory.

Conventional treatments for metabolic diseases are challenged by the existence of metabolic memory. For instance, glycemic control using hypoglycemic drugs was previously believed to be the primary approach to managing T2DM and its complications.^13^ However, it has been discovered that despite achieving great glycemic control, the organism continues to exhibit various inflammatory responses and complications associated with diabetes due to metabolic memory.^14,15^ This realization prompts further in-depth investigation into the molecular mechanisms underlying metabolic memory, aiming to develop corresponding therapeutic interventions that can effectively mitigate or eradicate the adverse effects associated with metabolic memory. This will ultimately enhance the treatment efficacy of various metabolic diseases.^16^ Accumulating evidence suggests that multiple intricate molecular and cellular mechanisms serve to establish and maintain metabolic memory, including epigenetic regulation, glycosylation end products, oxidative stress, etc. These interconnected mechanisms form a complex network that governs metabolic memory and can emerge as novel targets for both detection and intervention of metabolic diseases.^17–19^

Here, we seek to outline the research history and distinct features of metabolic memory. We also summarize the various molecular and cellular mechanisms that regulate metabolic memory. Furthermore, we emphasize the existence and profound impact of metabolic memory in numerous metabolic diseases and establish connections between these mechanisms and disease progression. Additionally, we specifically focus on the significant research advancements linking metabolic memory with cancer risk. Moreover, we explore the potential utility of the phenomenon of metabolic memory and its associated mechanisms as indicators and promising targets for the detection and therapeutic interventions of metabolic diseases.

## The research history of metabolic memory

**Fig. 1:**
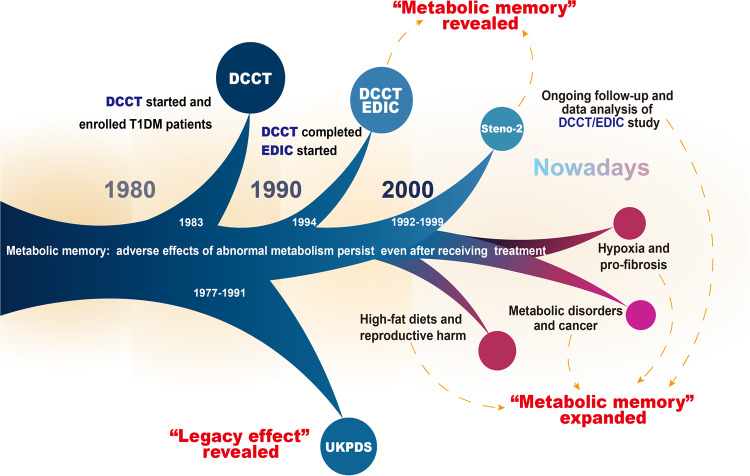
The research history of metabolic memory. The term “metabolic memory” originated from the study of the pathogenesis of long-term diabetic complications by the Diabetes Control and Complications Trial (DCCT) in 1983. In 1994, a long-term prospective, longitudinal, observational study conducted by Epidemiology of Diabetes Interventions and Complications (EDIC) found that the risk of complications in diabetic patients with regular glycemic control was higher in the conventional treatment group than in the early intensive control group. This phenomenon has been characterized as “metabolic memory” by the DCCT/EDIC. The following clinical trials, like UKPDS and Steno-2 trials, also revealed early intensive glycemic control might bring about prolonged benefits in diabetes care. Nowadays, the concept of metabolic memory and its implications have been expanded, especially in hyperglycemia, hyperlipidemia, hypoxia, and other metabolic disorders

“Metabolic memory” originated from studies of diabetes and its complications (Fig. 1). In 1983, the Diabetes Control and Complications Trial (DCCT) was initiated to investigate “glucose hypothesis” of diabetic complications, which suggested that hyperglycemia critically affected long-term diabetic complications.^20,21^ There were 1441 individuals with type 1 diabetes mellitus (T1DM) attending the DCCT by 1989.^22,23^ Participants were randomized to either the intensive therapy group or the conventional therapy group. The intensive therapy participants received a minimum of 3 daily insulin injections or continuous insulin infusion using external pumps, aiming to achieve tight glycemic control comparable to non-diabetic people.^24^ The conventional therapy participants received 1 or 2 daily insulin injections with the goal of safely achieving asymptomatic glucose control.^24^ In 1993, after the mean follow-up period of 6.5 years, the study results demonstrated that intensive therapy provided a significant reduction in the development and evolution of diabetic retinopathy, nephropathy, and neuropathy versus conventional therapy. Notably, almost all beneficial effects were statistically attributed to the difference in mean glycosylated hemoglobin A1c (HbA1c) levels.^25,26^ These findings strongly support the glucose hypothesis by emphasizing blood glucose levels are the primary driving factor behind the development of diabetic complications.^20,27^

After DCCT termination, a subsequent long-term observational follow-up study called the Epidemiology of Diabetes Interventions and Complications (EDIC) was initiated in 1994 and is still ongoing. The EDIC study involved 1394 surviving participants from the DCCT cohort.^28^ Individuals initially assigned to receive conventional treatment were subsequently exposed to intensive treatment, while every subject then returned to their respective healthcare provider to receive further treatment. During EDIC, HbA1c values in both original conventional and intensive treatment groups rapidly converged. Considering that mean HbA1c levels significantly influenced complication outcomes between intensive and conventional therapy groups over the period of DCCT, it would be reasonable to expect a similar trend in complication development between both treatment groups during EDIC. Surprisingly, though, the initial 4-year follow-up period of EDIC unveiled escalating disparities in complication rates between the two groups, with a notable reduction in retinopathy progression and nephropathy risk observed within the initially intensively treated group in comparison to the conventionally treated group.^29^ Results of the first 8-year follow-up further elucidated that earlier HbA1c levels largely influenced the long-term risk of complications and that the pathological alterations caused by hyperglycemia persisted after the hyperglycemic period, which was referred to as “metabolic memory”.^14^ Subsequent investigations and data analysis indicated that the biological impacts of metabolic memory peaked during the first decade and decayed thereafter.^11^ The findings of the DCCT/EDIC study and the discovery of metabolic memory highlight the great significance and prolonged benefits of early strict glycemic control, bringing about a dramatic change in diabetes management.

Comparable advantages have also been reported in the UK Prospective Diabetes Study (UKPDS) (referred to as the “legacy effect”) and the Steno-2 trial.^30–33^ During the period 1977–1991, 5102 newly diagnosed diabetic patients between 25 and 65 years old were recruited from 23 participating hospitals. Among them, 4209 eligible patients were randomly assigned for either the conventional or intensive glycemic control. The intensive therapy group exhibited a significant 25% risk reduction of microvascular lesions and a 16% risk reduction (*P* = 0.052) in myocardial infarction in the end.^30^ The UKPDS post-trial study, which followed patients with newly diagnosed T2DM on conventional and intensive therapy for up to 10 years, showed a significant reduction in endpoint events related to diabetes (including microvascular lesion, myocardial infarction, and death) in subjects receiving intensive therapy in comparison to conventional therapy. The findings imply that early intervention in blood glucose may make the most significant contribution to the prevention of T2DM complications.^10^ Furthermore, 160 participants with T2DM and microalbuminuria (with a mean age of 55) were randomized and allocated to undergo either conventional or intensive therapy in the Steno-2 trial, and they were followed for the mean 13.3-year duration.^34^ Despite the convergence of glycemic control after the end of the study, participants treated with intensive control experienced a decreased risk of cardiovascular incidents and cardiovascular and all-cause mortality.

Several recent research studies have expanded the concept of metabolic memory with its implications in various pathologic states. Similar to hyperglycemia, abnormal fat and cholesterol levels contribute to prolonged cellular alterations and tissue damage.^35,36^ For instance, a set of works by Crisóstomo et al. discovered the correlation between the early high-fat diet and irreversible alterations in testicular lipid content and metabolism. These changes appear to be related to lasting impairments in sperm quality in the future, and switching to a regular diet cannot reinstate the quality of sperm.^37,38^ This phenomenon, known as “inherited metabolic memory” caused by exposure to an elevated-fat diet, alters fatty acid metabolism in the testes with harmful effects on sperm that can last for up to two generations. “Inherited metabolic memory” is reported to be associated with sperm small non-coding RNAs (sncRNAs) content.^12,39,40^ The high-fat diet alters the accessibility of mice liver chromatin, with a substantial proportion of loci remaining altered after the diet returns to normal. These long-lasting chromatin accessibility changes were discovered to be correlated to specific transcription factors as well as histone modifications, indicating that long-term risk of metabolic diseases may be impacted by persistent epigenetic modifications induced by high-fat diets.^41^ A recent study of the medaka fish also illustrated that early nutritional conditions may consistently influence the animal’s metabolism. The study found that the medaka fish fed with high-fat food during early life developed hepatic steatosis with substantial hepatocyte gene expression alternations. Prolonged normal feeding reversed a majority of epigenetic modulations induced by the previous high-fat diet, whereas some loci around genes associated with hepatofibrosis and hepatocarcinogenesis still showed non-reversible changes.^42^

Long-term pathological alterations are likewise mediated by abnormal metabolic reprogramming in hypoxia. It was found that fibroblasts in hypoxic environments are also capable of generating metabolic memory. Hypoxia-induced pulmonary hypertension causes fibroblasts to undergo metabolic reprogramming, shifting the metabolic paradigm toward aerobic glycolysis, accompanied by increased free nicotinamide adenine dinucleotide (NADH) and NADH/ nicotinamide adenine dinucleotide (NAD) ratios. Increased free NADH further activates C-terminal binding protein 1 (CtBP1), driving the proliferation and pro-inflammatory phenotype of fibroblasts in turn.^43^ Significantly, the same metabolic reprogramming event, along with enduring inflammation and fibrosis, was observed when these fibroblasts returned to normoxic culture conditions. Hypoxia also evokes the generation of metabolic memory in cardiac fibroblasts via inducing alterations of expression of DNA methyltransferase (DNMT) enzymes and develops a long-lasting pro-fibrotic milieu.^44,45^ Recent studies on hypoxia-mediated cell metabolic reprogramming in the tumor microenvironment have provided novel perspectives regarding cancer pathogenesis. Hypoxia has been recognized to be an important cancer hallmark and is positively associated with cancer progression, metastasis, and therapeutic resistance.^46^ Hypoxia-inducible factor-1α (HIF-1α) mediates the adaptive response of tumors to hypoxia and was found to be highly overexpressed in the majority of solid tumors and their metastases.^47–49^ It has been demonstrated that hypoxia can upregulate transcriptional activity and stability of HIF-1α expression through a range of epigenetic modifications and influences the expression of numerous epigenetic modulators in a manner dependent on HIF-1α.^50,51^ The persistence of transcriptional reprogramming induced by the hypoxic tumor microenvironment leads to upregulation of the glycolytic program and increased lipolysis, driving cancer cell proliferation, migration, and immune escape.^52–55^

In addition, a number of recent research studies suggest that high levels of uric acid could influence the immune response through persistent epigenetic modifications, resulting in an altered functional state of immune cells that persists after removing the initial stimulus. The methylation level of the C-C motif chemokine ligand 2 (CCL2) promoter is dramatically reduced in Chinese Han male gout patients.^56^ A recent DNA methylation sequencing of gout patients and healthy individuals showed differential DNA methylation of numerous genes in signaling pathways linked to innate and adaptive immunity as well as osteoclastogenesis, including interleukin 17 (*IL-17*), signal transducer and activator of transcription 2 (*STAT2*), interferon regulatory factor 1 (*IRF1*), and myocyte-specific enhancer factor 2 C (*MEF2C*), etc.^57^ Peripheral biological mononuclear cells (PBMC) from gout patients and PMBC from healthy subjects treated with uric acid produce enhanced levels of pro-inflammatory cytokines stimulated by toll-like receptor (TLR) agonist compared to controls and maintain a high response potential at stimulation intervals.^58^ Treatment with histone methyltransferase inhibitors reversed the persistent effects of urate. Another study reported that romidepsin, a histone deacetylase (HDAC) 1/2 inhibitor, reduced pro-inflammatory cytokine production in PBMC stimulated with monosodium urate (MSU) crystals.^59^ Long-term effects of uric acid-mediated epigenetic changes on hyperuricemic complications and targets for intervention require further studies.

In summary, metabolic memory, as a concept initially proposed in studies on diabetes and its complications, described possible adverse effects of short-term abnormalities in glucose metabolism on long-term health. Recent research on hyperglycemic memory revealed that hyperglycemia may lead to persistent complication progression even after glycemic control, suggesting the importance of early and strict control of hyperglycemia. The field of metabolic memory has also been expanded by many recent studies to encompass a number of metabolic activities except glucose metabolism, including lipid metabolism, oxygen metabolism, uric acid metabolism, and others, all of which may have far-reaching effects on the host through underlying mechanisms. In the review, we define metabolic memory as the ability of an individual to retain memory of the damage caused by aberrant metabolism that persists after normalization of metabolism rather than a separate description of long-term adverse effects or toxicity of glucose.

## The characteristics of metabolic memory

Metabolic memory refers to the distinct phenomenon in which detrimental impacts of a transient abnormal metabolic state on the body remain after normalized metabolism. Several basic experiments have previously provided valuable insights into some important characteristics of metabolic memory.

### Persistence

The first distinct hallmark is that metabolic memory promotes persistent harmful effects, including inflammatory changes, premature cell senescence, apoptosis, etc. Vascular smooth muscle cells (VSMCs) in diabetes models show remarkably enhanced expression of pro-inflammatory-related genes as well as associated inflammatory molecules.^60,61^ Interestingly, even after glucose normalization, VSMCs derived from diabetic mice continue to show elevated oxidative stress levels and enhanced inflammatory signaling pathway activation. This indicates that metabolic memory confers a pro-inflammatory phenotype on VSMCs, contributing to increased vascular dysfunction and atherosclerosis that occur in patients with diabetes. Furthermore, studies of human endothelial cells raised in high glucose concentration environments have observed prolonged and sustained upregulation of fibronectin gene expression, even when transferred to a medium with normal glucose. This is considered to be correlated to diabetic complications progression.^62^ Further research successfully replicated this metabolic memory phenomenon in animal models with diabetic nephropathy or retinopathy.^63–65^ These studies well implicated the association between metabolic memory and persistently aberrant expression of anti-oxidant and inflammatory genes.^66–69^

The abnormal metabolic microenvironments are well known to accelerate the senescence process in multiple cell types by causing mitochondrial dysfunction, increasing the generation of advanced glycation end products (AGEs) and reactive oxygen species.^70–73^ Prematurely senescent cells show heightened metabolic activity that enhances the release of proinflammatory cytokines, chemokines, as well as growth factors, collectively termed senescence-associated secretory phenotype (SASP). This leads to further development of inflammatory damage and establishes a harmful positive feedback loop in diabetes.^74,75^ Furthermore, studies have shown that the activity of secreting pro-inflammatory factors in senescent critical immune cells like macrophages remains upregulated after transient exposure to high glucose concentration, suggesting that metabolic memory promotes sustained cellular senescence and release of SASP factors.^76–78^ Metabolic memory also allows pro-apoptotic activation to persist despite termination of hyperglycemia.^79^ A series of apoptosis-associated genes, such as the tumor necrosis factor (TNF) receptor and ligand and the B-cell lymphoma-2 (Bcl-2) family, remained elevated in retinal cells after re-establishment of good glycemia management of diabetic rats. Finally, and notably, the duration of these adverse consequences mediated by metabolic memory varies depending on the source, extent, and duration of the stimulus. Most experimental results vary from study to study depending on the investigator’s protocol design. With respect to current in vivo and in vitro studies of glucose and lipid stimuli, the duration of metabolic memory after stimulus elimination is at least as long as the duration of the previously received stimulus.^62,66,80^

### Progressivity

The second hallmark of metabolic memory is the long-term adverse effects on metabolic complications, which depend on early control; subsequent metabolic control does not prevent progressive complications. One of the earliest associated studies published in 1987 compared and analyzed the incidence of retinopathy in dogs with poor glycemic control, good glycemic control, and good glycemic control after a period of poor glycemic control.^81^ The results demonstrated that the incidence of retinopathy in dogs with good glycemic control after poor glycemic control was similar to that in the poor glycemic control group and higher than that in the good control group. Moreover, by the completion of the trial, the severity of retinopathy in the last group was greater compared to the end of their period of poor glycemic control, indicating that subsequent control failed to prevent the continued diabetic complications progression caused by early hyperglycemia. Lack of intensive management in the early stage of diabetes can lead to prolonged, irreversible inflammatory responses and oxidative stress in tissues like kidneys and retina. The Action in Diabetes and Vascular Disease: Preterax and Diamicron Modified Release Controlled Evaluation (ADVANCE) and Veterans Affairs Diabetes Trial (VADT), comprising individuals with uncontrolled glycemia of long duration, found no significant benefit of intensive glycemic management on major cardiovascular disease incidents.^82,83^ These results suggest that the longer the duration of hyperglycemia, the less impact intensive glycemia control has on diabetic complications.^84^ Consequently, diabetic complications progression may perpetuate even when removed from the hyperglycemic environment.

Maternal high-fat dietary interventions also affect tissue immunity as well as metabolic homeostasis in offspring in a timing-dependent intervention.^85^ Compared with offspring from experimental groups in which maternal mice were switched from a high-fat diet to a normal-fat diet 9 weeks before pregnancy, offspring from experimental groups switched 1 or 5 weeks before pregnancy showed earlier and more severe glucose intolerance, hepatocyte degeneration and adipose tissue inflammation.^86,87^ Enhanced adipogenic genes and hyperactivation of inflammatory signaling pathways were found in these offspring, accompanied by reduced expression of insulin receptor substrates and blunted insulin signaling.^88^ Thus, the early state of metabolic control is critical for the long-term memory impacts conducted by metabolic disorders, and traditional interventions may not be effective in alleviating progressive complications. The reversibility of adverse effects depends on the timing of intervention and therapeutic measures.

### Epigenetic modifications regulation

Finally, recent studies have highlighted the close association between sustained detrimental influences of metabolic memory and epigenetic regulation. A series of studies by Kowluru et al. demonstrated that transient or prior hyperglycemia led to various persistent epigenetic modulations, including DNA methylation, histone methylation, and histone acetylation. These modulations lead to sustained activation of pro-inflammatory pathways as well as oxidative stress.^68,80,89,90^ Consequently, these epigenetic modifications contribute to the enduring adverse effects of early abnormal metabolic conditions on cellular functions, perpetuating the pro-inflammatory and pro-destructive metabolic memory and driving diabetic complications progression after subsequent normoglycemia. As is similar to the long-term effects mediated by hyperglycemia, the short-term of a high-fat diet leads to disruption of the expression of key markers correlated with the regulation of cholesterol and lipid metabolism, with long-term adverse effects mediated by persistent epigenetic modifications.^91,92^ As previously described, epigenetic modifications in the liver were found in the early-life high-fat diet mice and would be maintained to varying degrees after resumption of a normal food diet.^41^ In addition, high-fat nutritional status in early life triggers irreversible epigenetic changes at specific gene locus in medaka, which are primarily histone modifications of the acetylation of lysine 27 on histone 3 (H3K27ac) and the methylation of lysine 9 on histone 3 (H3K9me3).^42^ ATAC-seq analyses identified multiple genes associated with hepatic fibrosis and hepatocellular carcinoma to show sustained gene signaling changes, including Ephrin type-A receptor 5 (*epha5*), raft linking protein (*raftlin*), and HERV-H LTR-associating protein 2 (*hhla2b1*), which may lead to an increased propensity for liver inflammation, fibrosis, and carcinoma. Maternal high-fat diets lead to persistent alterations in hepatic DNA methylation and histone modifications in offspring fetuses, increasing susceptibility to metabolic syndrome and steatohepatitis.^93,94^ To investigate the regulation mechanisms of epigenetic changes in metabolic memory maintenance, various animal models, including atherosclerosis, diabetic nephropathy, diabetic retinopathy, and other diseases, have been established. These disease models yield valuable insights into the intricate relationship between epigenetic modification and the persistence of metabolic memory.

**Fig. 2:**
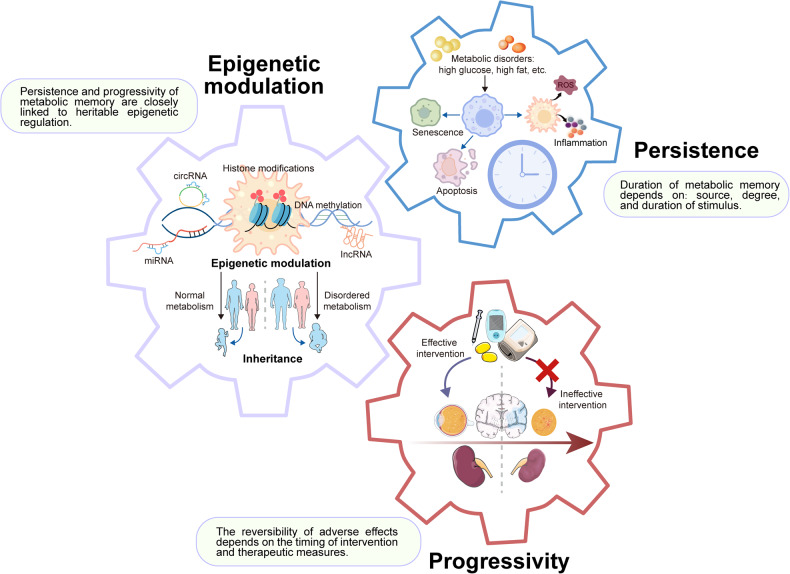
The characteristics of metabolic memory. Metabolic memory has three distinct hallmarks. Firstly, the long-term adverse effects on diabetic complications depend on early glycemic control, as subsequent glycemic control does not prevent progression. Secondly, metabolic memory promotes inflammatory changes, premature cell senescence, and ongoing apoptosis, perpetuating the harmful effects even after hyperglycemia is resolved. Thirdly, the establishment of metabolic memory is highly associated with epigenetic modifications, contributing to the enduring adverse effects and progression of diabetic complications. The figure was created with the assistance of Servier Medical Art (https://smart.servier.com/)

In summary, metabolic memory refers to the phenomenon where adverse impacts of transiently abnormal metabolic conditions persist even after metabolic normalization. It has three distinct hallmarks (Fig. 2). Firstly, metabolic memory promotes persistent harmful effects including inflammatory changes, premature cell senescence, apoptosis, etc. Secondly, the long-term adverse effects of metabolic complications depend on early control, while subsequent metabolic control does not prevent progressive complications. Thirdly, the establishment of metabolic memory is highly correlated to epigenetic regulation, resulting in the enduring adverse effects and progression of metabolic complications, which may be transmitted to offspring.

## Molecular mechanisms of metabolic memory

**Fig. 3:**
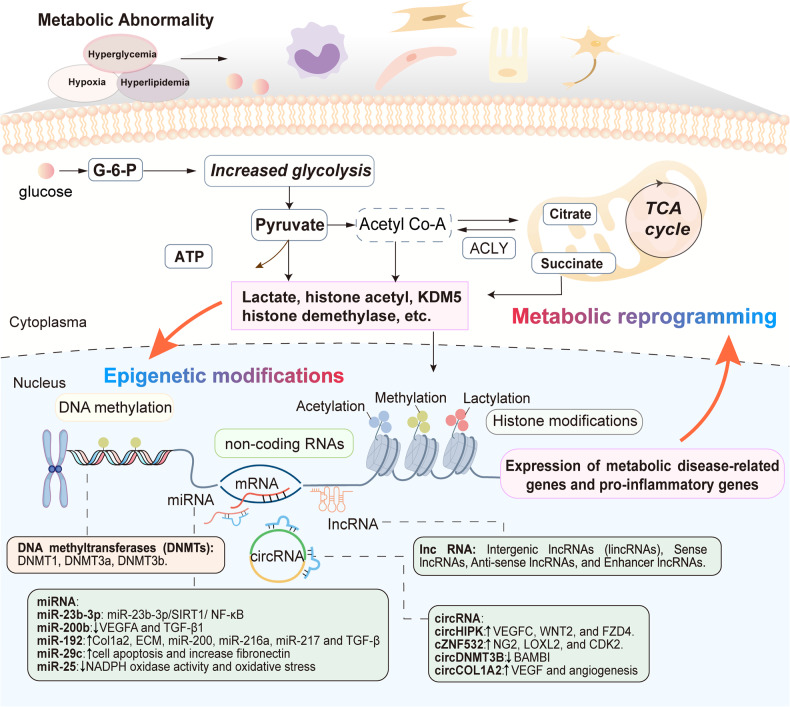
An overview of the interplay between epigenetic modifications and metabolic reprogramming during metabolic memory. The molecular mechanisms of metabolic memory mainly include epigenetic modifications and metabolic reprogramming. Accumulation of metabolic intermediates induces epigenetic modifications, including DNA methylation, histone modifications, and non-coding RNAs (ncRNAs). DNA methylation and histone modifications take place at the level of chromatin, while ncRNAs modulate gene expression mainly at post-translational level. Epigenetic modifications could induce persistent expression of metabolic disease-related genes and pro-inflammatory genes, which interact and work together

Mounting evidence has emerged indicating a close association between epigenetic modifications and metabolic memory in recent years. Epigenetic alterations have been detected in diverse target cells when exposed to disrupted metabolic circumstances, and these changes persist after metabolism levels return to normal, suggesting that epigenetic modifications may serve as the underlying molecular mechanism for metabolic memory.^95–98^ Chromosomes consist of DNA–protein complexes known as chromatin, which are composed of nucleosomes as their subunits.^99^ Each nucleosome consists of a complex of octameric histone, composed of dimers of core histones H2A, H2B, H3, and H4, intricately enveloped by 147 base pairs of DNA. Specific gene expression is regulated by epigenetic modifications without altering the original DNA sequence.^100^ These different modifications occur at distinct levels of nucleic acids and histones, encompassing DNA methylation, modifications to histones, as well as non-coding RNAs (ncRNAs), working synergistically to govern gene function and expression.^101^ In general, altered metabolic circumstances lead to the initiation of cellular metabolic reprogramming, resulting in changes in metabolites that subsequently impact epigenome-modifying enzymes that use intermediate metabolites as substrates.^102^ These regulatory processes, mediated by epigenetic changes, enable cells to respond rapidly to ever-changing environmental stimuli and acquire long-term responsiveness even when the initial stimuli are removed (Fig. 3).^103,104^

### DNA methylation

DNA methylation is a prevalent biochemical process that involves the addition of methyl groups to DNA molecules. In mammals, the process of DNA methylation occurs primarily at dinucleotides consisting of cytosine-phosphate-guanine (CpG), leading to the formation of 5-methylcytosines, although non-CpG methylation may also occur.^105^ The CpG-rich regions are predominantly situated in regulatory domains and play an instrumental role in gene transcription. DNA methylation in promoter regions typically suppresses gene expression by hindering the interaction between transcription factors or by enlisting chromatin inactivation complexes in conjunction with methyl-CpG binding domain proteins, leading to the suppression of transcription. On the other hand, methylation occurring in the gene’s body could impact both transcription elongation and alternative splicing.^106^ The process of DNA methylation is significantly influenced by the crucial involvement of DNMTs. DNMT1 is responsible for recognizing and methylating CpG islands located on the newly formed DNA strand, thereby transmitting epigenetic information across cell generations to maintain methylation. Meanwhile, the initiation of de novo DNA methylation is attributed to DNMT3a and DNMT3b.^107,108^ The process of DNA methylation is a dynamic modification that can be reversed by inhibiting the activity of DNMT1 and activating DNA demethylases, such as the ten-eleven translocation (TET) methylcytosine dioxygenases, which actively eradicate the methyl group from 5-methylcytosines.

Several experimental and clinical studies have presented convincing findings supporting the correlation between DNA methylation and the persistence of metabolic memory. Previous investigations have demonstrated that environment factors and dietary choices may affect epigenetic modifications, there contributing to individuals’ vulnerability to metabolic diseases.^109,110^ Analysis of DNA methylation in blood/DNA samples obtained from participants in the DCCT/EDIC study has revealed a significant correlation between long-term preceding glycemic history and DNA methylation changes. A total of 186 CpGs were identified as being associated with the average level of HbA1c in DCCT.^111^ Importantly, the majority of these HbA1c-associated CpGs exhibited significant enrichment within enhancers or transcription-related regions of blood cells and hematopoietic stem cells, particularly at CCAAT/enhancer binding protein (C/EBP) binding sites. C/EBPs exert a vital role in governing hematopoiesis and the differentiation of blood cells and are associated with oxidative stress and inflammation in both blood cells and target cells involved in diabetic complications.^112^ Changes in the DNA methylation patterns at CpG sites associated with HbA1c have an impact on hematopoietic cells and other target cells, promoting immune response and inflammation and ultimately contributing to disease development. These results highlight the significance of DNA methylation at specific CpG sites in the progression of complications associated with diabetes. Notably, the persistence of DNA methylation differences at HbA1c-associated CpGs further strengthens the link between DNA methylation and metabolic memory.^113^

Various reports have documented different methylation levels of genes associated with diabetes. Differential methylation levels were found between individuals with T1DM and healthy subjects at four CpG loci near the insulin gene encoding pre-insulin. Specifically, CpG-19, 135, and 234 exhibited hypomethylation, whereas CpG-180 displayed hypermethylation, all of which were associated with an elevated susceptibility to the development of T1DM.^114^ Similar results have been documented in research investigating specific complications. The global levels of DNA methylation exhibited a significant increase in individuals with T2DM who presented albuminuria, as compared to those without albuminuria. Moreover, a positive association was observed between the severity of albuminuria and the identified increased levels.^115^ Furthermore, genome-wide analysis of DNA methylation in DNA derived from peripheral blood cells of individuals diagnosed with T1DM, both with or without diabetic nephropathy, unveiled the discovery of 19 CpG loci linked to the susceptibility to diabetic kidney disease.^116^ A higher methylation level of T1DM patients was observed at a specific CpG island positioned upstream of the transcriptional start site of the unc-13 homolog B (*UNC13B*) gene, which was previously been associated with the development of diabetic nephropathy.^116^ In addition, high glucose also induces activation and overexpression of DNMT1 and promotes apoptosis and oxidative stress through DNMT1-mediated methylation of peroxisome proliferator-activated receptor α (PPARα), leading to exacerbation of diabetic retinopathy.^117^ Elevated expression of DNMT1 in histiocytes was found in diabetic mice, as well as in peripheral immune cells of diabetic patients. This upregulation is associated with the activation of multiple inflammatory pathways.^118,119^

More importantly, it has been discovered that altered methylation levels mediated by early metabolic abnormalities are not reversed as metabolism returns to normal. In the retinas of diabetic rats induced by streptozotocin, there was an increase in methylation levels within the promoter region of polymerase gamma (POLG1), which is responsible for encoding the catalytic subunit of mitochondrial DNA replicase.^89^ The hypermethylation was observed even after glucose levels were restored to their normal range. Similar results were noted in the retinal endothelial cells that were exposed early to high glucose.^89^ On the contrary, a decrease in global DNA methylation was noted in fibroblasts from diabetic foot ulcers when compared to fibroblasts from non-diabetic feet.^120^ This DNA methylation pattern remained consistent across multiple cell cultivation sessions under normoglycemic conditions. Moreover, studies conducted on diabetic zebrafish and rats induced by STZ also demonstrated a chronic hyperglycemia-induced overall DNA hypomethylation that perpetuated under normoglycemic conditions.^121,122^

Short-term high-fat diets have demonstrated the ability to induce long-term modifications in DNA methylation. Kim et al. found that a regular diet administered for 9 weeks after a high-fat diet of the same duration was able to reverse the non-alcoholic fatty liver disease phenotype, but elevated serum triglyceride levels and changes in gut microbiome composition persisted.^123^ Analysis of the changes in microbiome composition revealed a persistent enrichment of *Odoribacter*, which is known to produce butyrate with histone deacetylation inhibitor effects.^124,125^ Further genome-wide DNA methylation studies have revealed persistent alterations in methylation patterns at loci associated with lipid and cholesterol metabolism, such as hypomethylation of the apolipoprotein A4 (*Apoa4*) gene, which is considered to contribute to elevated triglyceride transport from liver to serum. Offspring exposed to a maternal high-fat diet are more susceptible to hepatic steatosis and inflammatory responses, with sustained changes in DNA methylation levels of key genes relevant to tissue development, metabolism, and cellular adhesion and communication. These genes include fibroblast growth factor 21 (*Fgf21*), peroxisome proliferator-activated receptor γ coactivator 1-beta (*Ppargc1β*), and von Willebrand factor (*VWF*), among others.^94^

Taken collectively, these findings propose that aberrant metabolic stimuli can trigger alterations in promoter methylation and exert a persistent impact on the expression levels of genes associated with oxidative stress, mitochondrial dysfunction, and apoptosis. This intricate process may contribute significantly to the establishment of metabolic memory.^126^

### Histone modifications

Histone modifications are a range of post-translational modifications that occur on specific residues of the N-terminal amino acids of histones. These modifications include but are not limited to acetylation, methylation, phosphorylation, ubiquitination, and others.^127^ These modifications can regulate the interplay between histones and DNA as well as other nuclear proteins, thereby either inhibiting or activating gene transcription. The impact on gene expression is contingent upon the type and degree of modification, along with the specific location of the altered amino acid residues.^128^ Different levels and types of histone modifications collaboratively facilitate epigenetic regulation to affect cellular metabolism by controlling the expression of relevant genes through intricate and diverse mechanisms.

Acetylation represents a highly dynamic process mediated by histone acetyltransferases (HATs), such as p300 and CREB-binding protein, along with HDACs encompassing HDAC1–11 and sirtuins. These enzymes are pivotal for orchestrating chromatin remodeling events.^99^ On one hand, the acetylation of lysine residues in the histone tails results in the reduction of their positive charge, thereby decreasing the binding affinity between histones and negatively charged DNA. This impedes the interplay between DNA and histones, facilitating chromatin opening and promoting gene transcription. What’s more, acetylation can contribute to the recruitment of specific transcription factors and cofactors that further augment expression levels of genes.^99^ Histone acetylation at gene promoters (e.g., H3K9ac, H3K14ac, and H3K56ac) is generally linked to the activation of transcription, while the elimination of acetyl groups is associated with histone condensation and gene repression.^129^ Hyperglycemia promotes the activation of HATs, leading to the acetylation of lysine residues on histones located in the promoter regions of proinflammatory genes, thereby amplifying the expression of inflammatory factors.^107,130^ For instance, retinal capillary endothelial cells cultured in hyperglycemic conditions exhibited a reduction in both expression and functionality of Class III HDAC sirtuin 1 (SIRT1). This decrease persisted even following the resolution of hyperglycemia, implying a correlation between metabolic memory and histone acetylation.^131^ High glucose levels lead to the inhibition of SIRT1, resulting in increased acetylation of target genes, including forkhead box o1 (*Foxo1*) and nuclear factor kappa B (NF-κB) subunit *p65*, ultimately leading to heightened oxidative stress and inflammatory responses.^132^ Additionally, it was observed that the expression of HDAC3 and HDAC4 is enhanced under high glucose conditions, exacerbating inflammation and fibrosis.^133,134^ Besides, histone acetylation on DNMT proteins could potentially contribute to the regulation of DNA methylation.^135^ Specifically, H3K9 acetylation of the DNMT1 promoter can activate DNMT1 by downregulating SIRT1.^136^

The impact of histone methylation on gene transcription encompasses various aspects, as it can either facilitate or inhibit gene transcription based on the modified amino acid residues and level of methylation. Transcriptional activation is linked to four methylation sites on histones: H3K4me1/2/3, H3K36me2/3, H3K48me3, and H3K79me3. Conversely, H3K9me3, H3K27me3, and H4K20me3 are associated with transcriptional repression.^99,137^ Histone methyltransferases (HMTs) selectively transfer methyl groups from S-adenosyl-l-methionine to lysine or arginine residues in a highly specific manner, which can be reversed by histone demethylases (HDMs).^138^ Multiple studies on diabetic rat models have revealed the reduced levels of H3K9me3 and H3K27me3, mediating the release of transcriptional repression at the promotor/enhancer regions of genes associated with fibrosis and inflammation. The upregulation of these genes, including *Il-6*, monocyte chemotactic protein-1(*Mcp-1*), collagen type 1 alpha 1(*Col1a1*), and plasminogen activator inhibitor-1(*Pai-1*), ultimately lead to enhanced inflammation and disease progression.^97,139–141^ Conversely, markers associated with transcriptional activation, such as H3K4me1, were found to be upregulated at the promotor site of *p65* in a high glucose environment and persisted after normalization of glucose levels.^66,67^ Mice subjected to an 8-week high-fat diet followed by an 8-week normal diet showed persistent lipid accumulation and elevated triglyceride levels. Formaldehyde-assisted isolation of regulatory elements sequencing (FAIRE-seq) analysis indicated persistent changes in the accessibility of chromatin for transcription factors, such as hepatocyte nuclear factor 4alpha (HNF4α), which were correlated with increased specific repressive histone modifications. Enrichment of H3K9me2 was found at sites with reduced chromatin accessibility.^41^ Similar to histone acetylation, histone methylation also modulates DNA methylation by affecting DNMT proteins.^142^ The methylation of H3K4 initiates de novo DNA methylation, leading to the activation of transcription. Conversely, transcription repression occurs as a result of the interaction between DNMT and H3K4 at the promoter level. Moreover, the recruitment of ubiquitin-like containing PHD and RING finger domains 1 (UHRF1) proteins by H3K9 with DNA methylation enhances the binding affinity of DNMTs to DNA, thereby promoting transcriptional repression.^143,144^

Histone phosphorylation can also influence histone–DNA interactions by altering the change of histones. The process of histone phosphorylation primarily occurs on serine, threonine, and tyrosine residues. A recent investigation unveiled an elevation in H3Ser10 phosphorylation within glomerular endothelial cells derived from diabetic patients, which mediates the amplified vascular cell adhesion protein 1 (VCAM-1).^145^ VCAM-1 promotes leukocyte adhesion and migration on the endothelium, thereby correlating with the advancement of diabetic nephropathy.^146^ Histone ubiquitination is typically observed at specific lysine residues located in the C-terminal tail of both histone H2A and histone H2B.^147^ The presence of high glucose levels induces H2A ubiquitination while reducing H2B ubiquitination, which activates the transforming growth factor-beta (TGF-β) pathway and accelerates disease progression.^148^ In the past few years, there has been a growing interest in studying histone lactylation as it plays a significant role in influencing gene transcription and metabolic regulation.^149^ This emerging field calls for further exploration into the association between histone lactylation and metabolic disorders, which holds significant potential for advancing our understanding in this area.

### Non-coding RNAs

Non-coding RNAs (ncRNAs) are a type of RNA molecule that does not possess the ability to produce proteins. Instead, they play a part in regulating gene expression at both the post-transcriptional and translational stages.^150^ ncRNAs can be primarily classified into two categories: structural RNAs, including rRNAs and tRNAs, and regulatory RNAs. The regulatory RNA group consists of sncRNAs, which have a length below 200 nucleotides (nt), and long ncRNAs (lncRNAs), which possess a length above 200 nt.^151^ The sncRNAs subgroup further comprises microRNAs (miRNAs), small interfering RNAs (siRNAs), PIWI-interacting RNAs (piRNAs), and other sncRNAs. The lncRNAs can be categorized based on their origin into intergenic lncRNA, bidirectional lncRNA, sense/antisense lncRNA, intronic lncRNA, and enhancer lncRNA.^152,153^ Recent evidence has provided new evidence indicating that the control of gene expression and modulation of growth factors and inflammatory factors associated with metabolic diseases can be influenced by both miRNAs and lncRNAs.

miRNAs are a group of endogenously encoded non-coding RNAs that are typically characterized by their short length, usually ranging from 17 to 25 bp. They operate by specifically binding to the mRNA of genes that encode proteins through base pairing, ultimately causing either degradation or inhibition after transcription.^154^ A single miRNA possesses the capacity to regulate numerous target genes implicated in diverse pathogenic pathways in metabolic memory.^155–157^ Conversely, it is also possible for a specific target gene to be modulated simultaneously by multiple miRNAs.^158^ The dysregulated expression of various miRNAs is responsible for the development of metabolic complications by impacting crucial pathological mechanisms, including angiogenesis, apoptosis, inflammation, and oxidative stress.^159,160^ For instance, in a hyperglycemic environment, there is an upregulation of miRNA-21 (miR-21) expression, which activates TGF-β and NF-κB signaling pathways, leading to inflammatory responses and apoptosis.^161^ Therefore, these important miRNAs exhibit potential as diagnostic and prognostic biomarkers, along with being prospective therapeutic targets for metabolic diseases and their associated complications in the future.^162^

LncRNAs, which are transcribed by RNA polymerase II or III and resemble protein-coding mRNAs, belong to a group of transcripts that cannot be translated.^163,164^ They are typically observed at minimal levels under normal circumstances but play crucial roles in regulating vital cellular physiological activities, including cell proliferation, differentiation, and senescence.^165,166^ However, their aberrant expression is strongly associated with the advancement of specific diseases.^167–169^ Recent investigations have revealed that lncRNAs actively participate in the regulation of several metabolic disorders through their influences on epigenetic modifications, transcriptional regulation, and post-transcriptional modulation.^170^ For instance, the lncRNA maternally expressed gene 3 (MEG3) has been reported to be downregulated in the retina of mice with STZ-induced diabetes, which exacerbates retinal microvascular dysfunction by activating phosphatidylinositol 3-kinase (PI3K)/protein kinase B (Akt) signaling pathway.^171^ Further investigation showed that the decrease in MEG3 expression is controlled by DNMT1-mediated methylation occurring at the promoter region of MEG3, thereby expediting the progression of endothelial-mesenchymal transition (endMT) in individuals with diabetic retinopathy.^172^

## Cellular mechanisms in metabolic memory

Numerous studies have presented evidence supporting the involvement of various cellular mechanisms in metabolic memory. These mechanisms, encompassing oxidative stress, non-enzymatic glycation of proteins, and low-grade inflammation, operate in a cascade and mutually reinforce each other, contributing to the persistence and progression of the deleterious effects of aberrant metabolism on the organism. This ultimately leads to abnormalities in cellular structure and function as well as organ pathology. Furthermore, environmental changes triggered by inflammation and oxidative stress further induce aberrant intracellular epigenetic modifications. These modifications subsequently boost the activation of genes associated with inflammation and programmed cell death, establishing a positive feedback loop that collectively sustains metabolic memory.^173^

### Oxidative stress

The initial findings by Brownlee et al. elucidated the generation of reactive oxygen species (ROS) is excessively enhanced, serving as a distinguishing feature in hyperglycemia-related reactions to various pathological states.^174^ Under hyperglycemic conditions, the electron transport chain in the tricarboxylic acid (TCA) cycle of metabolism experiences an increase in electron donors like NADH and flavin adenine dinucleotide (FADH2), resulting in an elevation of the voltage gradient across the mitochondrial membrane. The elevated voltage gradient promotes excessive generation of superoxide (O_2_^−^) and reactive oxygen species (ROS).^175,176^ Meanwhile, elevated blood sugar levels increase the production of diglycerides (DAG), triggering protein kinase C (PKC) activation and increasing NADPH oxidase activity. Consequently, this results in an augmented generation of O_2_^−^. ROS, such as peroxynitrite (ONOO^−^), readily penetrate cell membranes, disrupting a variety of intracellular structures and contributing to nuclear and mitochondria DNA destruction and damage.^177–179^ It should be noted that mitochondrial DNA is highly sensitive to oxidative stress, causing both structural and functional impairments within the mitochondria. This damage further triggers cumulative ROS, perpetuating a detrimental cycle.^180^ Sustained overproduction of ROS can explain hyperglycemic metabolic memory even after normalization of glycemic level.^181^ Notably, despite the short half-life of excess free ROS, their presence endures after the normalization of blood glucose, contributing to the memory phenomenon and cell-damaging effects.^63,65,182^ These detrimental effects of ROS can be antagonized by broad-spectrum antioxidants acting at the mitochondrial level.^19,183^ In addition, it has been observed that an excessive amount of ROS can drive a range of pathological cellular mechanisms, including heightened polyol and hexosamine fluxes, AGEs, as well as NF-kB-induced vascular inflammation.^184,185^

### Advanced glycation end products

AGEs, a diverse group of glycosylated adducts generated through a complex “Maillard reaction”, can be produced both internally within the body and externally from outside sources.^186,187^ The primary origin of AGEs is the endogenous process, whereby sugars react with amino groups of proteins, lipids, and nucleic acids through a series of non-enzymatic reactions.^188^ The physiological formation and accumulation of AGEs occur naturally during aging; however, insulin resistance, inflammation, and oxidative stress expedite this progression while also mediating cytopathic changes such as inflammation, fibrosis, and thrombotic reactions.^189–192^ The levels of AGEs have been found to be significantly higher in patients with MAFLD, diabetes mellitus, and its complications. These AGEs have also been linked to the risk of disease progression and mortality.^193–197^ Notably, due to their resistance to degradation, proteins modified by AGEs persist in the blood vessels, kidneys, and heart of diabetic patients even after achieving glycemic control. Thus, AGEs may exert important roles in metabolic memory.^18,198^

The deleterious impacts of AGEs could be attributed to three primary molecular mechanisms: modification of extracellular proteins, alteration of intracellular proteins, and activation of signaling cascades by binding to the receptor for AGE (RAGE) situated on the cell surface.

#### Modification of extracellular proteins

AGEs induce structural and functional abnormalities in tissues through the modification of extracellular proteins, resulting in the formation of stable and anomalous crosslinks. Specifically, collagen, laminin, and other extracellular matrix proteins can be altered through glycation by AGEs, leading to the formation of abnormal crosslinks that are resistant to proteolytic digestion.^199^ This process ultimately leads to the thickening of the basement membrane in vascular endothelial cells to impact the thickness and flexibility of blood vessel walls.^200–202^ In addition, AGEs can induce glycation in circulating factors like fibrinogen, low-density lipoprotein (LDL), etc., resulting in an intensified response to blood clot formation and an increased tendency for blood coagulation. Studies have shown that glycated LDL reduces tissue plasminogen activator (tPA) production in vascular endothelial cells and enhances thrombosis.^202,203^ Furthermore, the accumulation of AGEs resulting from hyperglycemia triggers the glycation and aggregation of lens α-crystallin. This process ultimately results in a decrease in lens clarity and an elevation in light dispersion, both of which are crucial in the development of diabetic cataracts.^204^

#### Modification of intracellular protein

AGEs trigger cellular damage through cross-linking or modifying various intracellular molecules, leading to the accumulation of improperly folded proteins within the endoplasmic reticulum (ER) through advanced glycation-mediated modification of molecular chaperones.^205^ This process triggers ER stress and disrupts cellular homeostasis.^206,207^ The persistence of ER stress and improperly folded proteins activates the unfolded protein response (UPR), ultimately leading to apoptosis.^208,209^ In addition, intracellular AGEs are able to bind to the mitochondrial respiratory chain complexes I and IV, which are involved in electron transport, and reduce their activity, decreasing ATP levels while increasing the production of superoxide and ROS, thereby inducing mitochondrial dysfunction.^205,210,211^ Furthermore, AGEs also diminish the activities of antioxidant enzymes like superoxide dismutase (SOD), catalase, glutathione peroxidase, and glutathione reductase. This acceleration further perpetuates the vicious cycle of ROS generation along with the accumulation of AGEs.^194,212^

#### AGE-mediated signaling cascades

The binding of AGE to RAGE is currently considered the primary pathogenic mechanism. RAGE, a pattern-recognition receptor belonging to the immunoglobulin superfamily, exhibits recognition and binding capabilities toward various ligands, including AGEs, Amyloid β (Aβ), S100/calgranulin, and the high mobility group box (HMGB)-1.^213,214^ The interactions between AGEs and RAGE trigger a series of intricate signaling cascades that culminate in producing pro-inflammatory factors and reactive oxygen species, ultimately fostering inflammation and tissue damage.^215^ It has been found that AGE-RAGE signaling triggers multiple downstream pathways, including mitogen-activated protein kinases (MAPK), AMP-activated protein kinase (AMPK), extracellular-signal-regulated kinases (ERK), and activates the NF-κB pathway, leading to a range of overexpressed cytokines and adhesion molecules, like IL-6, TNFα, TGF-β, and VCAM-1, etc.^212,216,217^ Deficiencies in RAGE lead to suppression of immune cell recruitment and attenuation of inflammation.^218^ RAGE activation also enhanced Janus kinase (JAK)/STAT signaling pathway activity and upregulated interferon-responsive gene expression.^219^ Since the promoter region of the RAGE gene contains an NF-κB-binding structural domain, interactions between AGE and RAGE interactions can elicit an upregulation in RAGE expression by enhancing inflammatory responses, which creates a positive feedback loop.^218^ Moreover, activation of AGE-RAGE has been observed to consistently upregulate NADPH and nitric oxide synthase, causing impaired mitochondrial function and elevated levels of oxidative stress.^219^ Sustained inflammatory responses and oxidative stress induce cellular fibrosis as well as apoptosis, which ultimately leads to vascular inflammation and diabetic vascular complications.^198,220,221^

### Low-grade inflammation

Low-grade inflammation is a significant determinant that potentially plays an integral part in metabolic memory. Inflammation has been identified as the major contributor to diabetes mellitus and its vascular complications. Extended inflammatory responses may act as agents responsible for metabolic memory. Reactive oxygen species (ROS) production is enhanced by metabolic reprogramming in response to modifications in the metabolic environment, which is crucial in linking epigenetic changes and the external surroundings, resulting in the upregulation of inflammatory factor expression and secretion. Enhanced inflammatory responses drive diverse epigenetic modifications in cells to adapt to environmental alterations. A positive feedback loop involving ROS, metabolic end products, inflammation, and epigenetic modifications leads to the progression of metabolic disorders.^222–224^ It has been well elucidated that activation of NF-κB is particularly instrumental in facilitating pro-inflammatory gene expression. In individuals with diabetes, inflammatory gene expression can be enhanced through the activation of NF-κB. The activation results in an increase of inflammatory cytokines associated with vascular inflammation, thereby promoting the synthesis of endothelial adhesion molecules, proteases, and other mediators. Monocytes are recruited and adhere to endothelial cells and vascular smooth muscle cells, ultimately leading to differentiation into macrophages.^225^ Toll-like receptors are also of great importance in connecting inflammation and oxidative stress, being identified as a pathogenic contributor to obesity and insulin resistance.^223^

In conclusion, oxidative stress and AGEs are pivotal factors implicated in metabolic memory and complications development. Oxidative stress, characterized by an excessive production of reactive oxygen species, plays a vital role in cellular damage and perpetuation of harmful effects even after metabolism returns to normal. AGEs, formed through nonenzymatic reactions, modify extracellular and intracellular proteins, triggering inflammation and impairing normal cellular functions. These processes contribute to vascular stiffness, thrombosis, atherosclerosis, and other pathological mechanisms. The inflammation and oxidative stress are further intensified by the activation of NF-κB and the interaction between AGEs and the RAGE receptor. A comprehensive understanding of these processes is crucial for developing strategies to mitigate metabolic memory and prevent complications associated with metabolic disorders.

## Cells involved in metabolic memory

### Immune cells

**Fig. 4:**
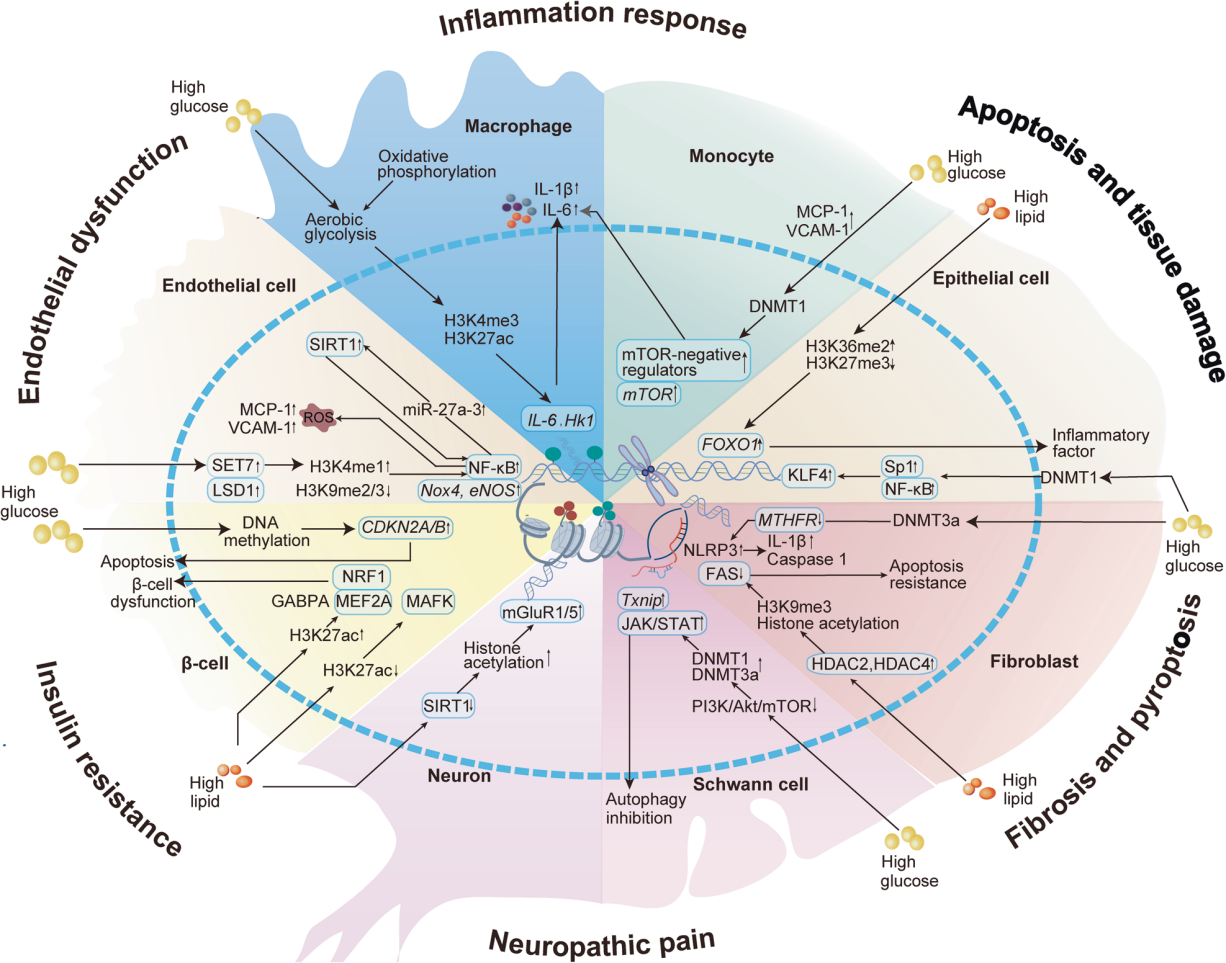
Different cell types involved in metabolic memory. Multiple types of cells are involved in the development of metabolic memory-mediated metabolic diseases and their complications. Different cells crosstalk with each other and work together to cause disease progression

Immune cells are key players in the formation and maintenance of metabolic memory. Hyperglycemia induces innate immune cells (e.g., monocytes and macrophages) to acquire metabolic memory and promote pro-inflammatory responses.^78,226^ Monocytes and macrophages are thought to undergo metabolic reprogramming and form metabolic memories in response to hyperglycemia, resulting in sustained activation that contributes to the pathogenesis of atherosclerosis.^227^ It was found that hyperglycemia can drive an increase in H3K4me3 and H3K27ac on the promoters of genes related to inflammation in macrophages and bone marrow-derived macrophages (BMDM) from diabetic mice through a glycolysis-dependent mechanism, thereby promoting proinflammatory gene expression (Fig. 4).^228^ Switching BMDM from a high glucose environment to physiologic glucose for culture did not alter the epigenetic and pro-inflammatory changes. In addition, normoglycemic mice received bone marrow transplantation from diabetic mice also exhibited increased aortic root atherosclerosis, suggesting a long-term effect of hyperglycemia mediated through metabolic memory.^111^ Histone-modifying enzymes are thought to upregulate MCP-1 expression and activate inflammatory monocyte differentiation as well as macrophage M1 polarization, leading to diabetic wound healing.^229,230^ In addition, hyperglycemia has been reported to upregulate DNMT1 expression to stimulate the mammalian target of the rapamycin (mTOR) pathway and induce pathogenic activation of peripheral blood mononuclear cells.^119^ Moreover, elevated lipid concentrations can also contribute to insulin resistance in monocytes, and this effect continues to persist even after lipid levels normalize due to metabolic memory. THP-1 monocytes treated with the saturated fatty acid, palmitate, for 6–12 h exhibited resistance to insulin-mediated glucose uptake and persisted for at least 60 h after removal of palmitate.^231^

### Endothelial cells

Endothelial cells are key players in mediating the link between vascular function and metabolic circulation.^232,233^ Metabolic disorders can modulate endothelial dysfunction and vascular diseases through metabolic memory.^234,235^ Sustained activation of p66^Shc^ by PKCβII has been observed in aortic endothelial cells of both humans and mice exposed to hyperglycemia, even after blood glucose has returned to normal.^236^ Activation of p66^Shc^ is associated with reduced CpG methylation and increased histone acetylation at promoters, which leads to elevated mitochondrial ROS production and oxidative signals translating into apoptosis.^237^ ROS, in turn, upregulate PKCβII activity, creating a detrimental cycle that ultimately culminates in apoptosis and the onset of tissue damage. A growing number of evidence has demonstrated that excessive ROS-mediated metabolic memory leads to a pro-inflammatory milieu and endothelial dysfunction in the aorta.^90,236,238^ NADPH oxidase 4 (Nox4) and endothelial nitric oxide synthase (eNOS) are involved in mediating transient hyperglycemia-induced vascular ROS generation and are regulated by altered histone methylation.^239^ An increase in H3K4me1 at the promoters of Nox4 and eNOS was found in endothelial cells in the hyperglycemic state, which antagonized and downregulated H3K9me2 and H3K9me3. Transient hyperglycemia facilitates the recruitment of lysine-specific methyltransferase SET7 and specific demethylase LSD1 to the NF-κB-p65 promoter. The upregulation of H3K4me1 by Set7 synergistically combines with the downregulation of H3K9me2 and H3K9me3 by LSD1 to activate the NF-κB pathway and upregulate the expression of inflammatory factors such as MCP-1 and VCAM-1, ultimately leading to dysfunction of vascular endothelium.^67,240^

Recent studies have also found that hyperglycemia-induced enhancement of the NF-κB pathway, increase in miR-27a-3p, decrease in nuclear factor erythroid-2-related factor 2 (NRF2) expression, and ROS overproduction in endothelial cells were maintained after restoration of normoglycemia, resulting in perivascular fibrosis and cardiovascular dysfunction in the heart.^241^ It confirmed the presence of metabolic memory in endothelial cells, indicating that insulin alone fails to improve cardiac function, whereas the combined application of miR-27a-3p inhibitors reverses the adverse effects. Moreover, studies on retinal endothelial cells have revealed that hyperglycemia can upregulate miR-23b-3p expression by activating the NF-κB signaling and target SIRT1 to mediate NF-κB acetylation, creating a positive feedback loop to maintain metabolic memory.^242^

### Epithelial cells

Metabolic memory is responsible for the accelerated accumulation of apoptosis and tissue damage in the epithelium. In mouse models with diabetic nephropathy, the reduction in the expression of Kruppel-like factor 4 (KLF4) was observed in podocytes, coinciding with the onset and exacerbation of proteinuria.^243^ The expression of KLF4 was associated with a decrease in methylation levels of renal unit promoters and promoters of other epithelial markers, simultaneously increasing the methylation of promoters of genes encoding mesenchymal markers, thereby reducing proteinuria. Further studies found that expression of DNMT1, NF-κB p65, and nuclear factor Sp1 was significantly increased in podocytes under diabetic state, promoting inflammatory responses and podocyte damage.^118^ The employment of DNA methylation inhibitors can downregulate DNMT1 expression and ameliorate the adverse effects of hyperglycemia through the Sp1/NF-κB p65-Dnmt1 pathway, attenuating albuminuria. In addition, podocyte apoptosis in diabetic nephropathy has been found to be closely associated with the regulation of various miRNAs.^244^ Disturbed lipid levels in circulation enhance FOXO1 activity and induce insulin resistance by modifying the abundance of H3K36me2 and H3K27me3 on the promoter region of FOXO1 in human urine-derived podocyte-like epithelial cells (HUPECs). These manifestations persist even after the normalization of circulating lipid levels.^245^

### Fibroblasts

Altered fibroblast function is strongly associated with delayed healing of diabetic wounds. Compared to nondiabetic donors, fibroblasts from T2DM donors exhibit changes in TNF-α-induced resistance to the inflammatory milieu, characterized by diminished synthesis of extracellular matrix (ECM), as well as impaired migratory and proliferative capacities. Such changes may be associated with chronic, nonhealing diabetic foot ulcers after restoration of normoglycemia.^246^ It was found that interferon-beta (IFN-β) stimulation allowed fibroblasts to acquire histone H3.3 and H3K36me3 chromatin marks, thereby establishing epigenetic memory.^247^ Genome-wide DNA methylation analysis of fibroblasts derived from diabetic foot ulcers also showed a significant decrease in global DNA methylation levels compared to non-diabetic foot fibroblasts.^120^ Fibroblasts of diabetic foot ulcer origin were identified as having sustained altered levels of DNA methylation even after prolonged passaging through normoglycemic conditions.^248^ Hyperglycemia also promotes diabetic cardiac fibrosis by regulating DNA methylation levels.^249^ 5, 10 methylenetetrahydrofolate reductase (MTHFR) is a crucial regulatory enzyme in cardiac fibroblasts that modulates fibrosis and pyroptosis.^250^ Significant increase of DNMT3a and decrease of MTHFR were observed in cardiac fibrosis tissues from both humans and mice with diabetes, accompanied by CpG hypermethylation of the MTHFR promoters. The enhanced pyroptosis mediated by MTHFR knockdown in cardiac fibroblasts was reversed upon knockdown of DNMT3a. Moreover, a high-fat diet could promote the resistance of lung fibroblasts to apoptosis by inhibiting the expression of the death receptor Fas (also called CD95), leading to the progression of pulmonary fibrosis.^251^ The Fas promoter in fibroblasts from the murine model of pulmonary fibrosis displayed a reduction in histone acetylation and an increase in H3K9me3, which correlated with elevated expression of HDAC2 and HDAC4.^252^ A high-fat diet has also been shown to induce H4K16ac accumulation, leading to pro-fibrotic gene overexpression and collagen deposition in lung fibroblasts.^253,254^

### Nerve cells

Neurons, Schwann cells, and glial cells are important participants in metabolic memory-mediated neuropathy.^255^ Metabotropic glutamate receptor (mGluR) 1 and mGluR5 are important proteins mediating neuropathic pain, with their expression significantly increased in diabetic neuropathic rats.^256^ The acetylation level of H3 in the promoter region of the gene encoding mGluR1/5 in spinal cord neurons was increased upon exposure to high glucose or high-fat stimulation, concomitant with a reduction in SIRT1 expression and activity. The SIRT1 activator, SRT1720, reversed the overexpression of mGluR1/5 and attenuated neuropathic pain in diabetic rats. Autophagy inhibition and dysfunction of Schwann cells are important pathogenic mechanisms in diabetic peripheral neuropathy (DPN). Reduced autophagy markers and brain-derived neurotrophic factor (BDNF) with increased expression of thioredoxin-interacting protein (TXNIP) in Schwann cells were observed in the sciatic nerves of diabetic mice.^257–259^ High glucose levels triggered the activation of the JAK/STAT signaling pathway, which induced HDAC1-dependent inhibition of autophagy and myelin abnormalities in Schwann cells.^258^ Expression of DNMT1 and DNMT3a in Schwann cells exposed to high glucose is regulated by the PI3K/Akt/mTOR pathway to mediate the progression of DPN.^257,259^

### Endocrine cells

Metabolic memory of endocrine cells is intimately linked to the continued progression of metabolic diseases. β-cells, crucial constituents of the pancreas, participate in insulin secretion upon hyperglycemia condition and facilitate glucose uptake by peripheral tissues.^260^ Dysfunction of β-cells serves as the central mechanism underlying diabetes development and is intricately linked with metabolic disorder-mediated epigenetic modifications.^261,262^ The results of several genome-wide DNA methylation sequencing of human islets from T2DM patients and healthy controls identified multiple genes associated with β-cell insulin secretion as being located in T2DM differentially methylated regions.^263,264^ Metabolic disturbances during pregnancy mediate the risk of T2DM in the offspring through CpG methylation modifications in insulin signaling-related genes.^265^ Hypomethylation in the promoter region of cyclin-dependent kinase inhibitor 2 A (CDKN2A/B) and pro-apoptotic genes has been reported in the offspring of rats with gestational diabetes, contributing to β-cell apoptosis and increased susceptibility to T2DM.^266^ In addition, obesity can also modulate β-cell function through activation of histone modifications. Mice fed with a high-fat diet exhibited an upregulation of H3K27ac in the binding regions of transcription factors such as NRF1, GA-binding protein transcription factor alpha subunit (GABPA), and MEF2A, while a downregulation of H3K27ac was observed in the binding region of MAFK, which is implicated in the negative regulation of β-cell function.^267,268^

## Metabolic memory and diseases

### Circulatory system

**Fig. 5:**
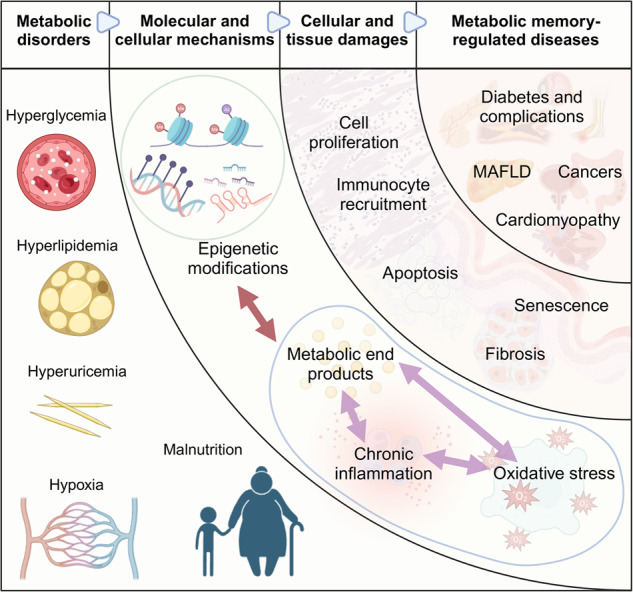
Complex interplay between metabolic memory and metabolic memory-regulated diseases. Metabolic disorders (including hyperglycemia, hyperlipidemia, hyperuricemia, hypoxia, and malnutrition) may induce epigenetic modifications and metabolic reprogramming at molecular and cellular levels, which take a toll on chronic inflammation and oxidative stress. Some metabolic end products, inflammatory cytokines, and reactive oxygen species could affect epigenetic regulations in return. Then, the epigenetic landscape and metabolic reprogramming could destroy the structure and function of different cells and tissues, manifesting as cell proliferation, immunocyte recruitment, cellular apoptosis, fibrosis, and senescence. The long-term accumulation of cellular and tissue dysregulation could give rise to metabolic memory-associated diseases, even after the elimination of metabolic disorders. The figure was created with BioRender.com (https://biorender.com/)

Diabetes significantly increases the incidence of cardiovascular diseases and mortality.^269^ Growing evidence suggests that metabolic memory is an essential factor contributing to the prolonged deleterious consequences of high glucose on the circulatory system (Fig. 5).^270^ The findings of the UKPDS showed that the deleterious influence of hyperglycemia on the microvascular and macrovascular complications in those with diabetes persisted after glycemic control.^10,31^ Hyperglycemia may increase methylation of the promoter region of sarcoplasmic/endoplasmic reticulum calcium-ATPase 2a (SERCA2a) by upregulating the expression of pro-inflammatory TNF-α.^271,272^ Decreased expression of SERCA2a leads to myocardial diastolic function disorder, thus triggering diabetic cardiomyopathy. Epigenetic modifications facilitated by hyperglycemia interfere with lengthy and relatively stable alterations in gene expression. For instance, aortic endothelial cells cultured in transient hyperglycemia show a sustained increase in H3K4me1 at the NF-κB p65 promoter, which persists under normoglycemia conditions.^66^ Transient hyperglycemia has also been shown to maintain hyperacetylation and mediate persistent endothelial senescence by regulating deacetylase and acetyltransferase activities in vascular endothelial cells.^273^ In addition, altered levels of some miRNAs have been proposed to be closely correlated to diabetic macrovascular complications, like miR-133a, miR-195-5p, and miR-457a, among others.^274–277^

Epigenetic modifications driven by metabolic environmental alterations promote the expression of inflammation- and fibrosis-associated genes, resulting in endothelial dysfunction.^278^ Endothelial dysfunction can induce functional impairment of the vasculature and heart by promoting the secretion of vasoconstrictor agents, elevated endothelial permeability, and pathologic angiogenesis.^279^ As previously described, high glucose can mediate a significant elevation of p66^Shc^ through decreased DNA methylation and increased histone acetylation, contributing to sustained oxidative stress and inflammation.^236^ p66^Shc^ activation can also upregulate miR-34a in endothelial cells, which can lead to increased p53 acetylation and apoptosis by targeting downregulation of SIRT1.^280,281^ Silambarasan et al. revealed a correspondence between the expression levels of several miRNAs and endothelial dysfunction with miRNA microarray analysis.^282^ Among them, miR-130b-3p, miR-140-5p, and miR-221-3p exhibited a positive correlation with endogenous glucose levels, triggering endothelial function disorders through targeting genes involved in inflammation, senescence, as well as apoptosis. Another study conducting ingenuity pathway analysis of miRNA variants in mice hearts with diabetes revealed that various dysregulated miRNAs are associated with transcriptional regulation of apoptosis, fibrosis, hypertrophy, and heart failure and would not be reverted by intensive glycemic control.^283^ In addition, microarray analysis of lncRNAs and circRNAs from endothelial cells in the hyperglycemic milieu also identified multiple changes of ncRNAs associated with vascular endothelial damage, further demonstrating that metabolic disorders epigenetically modulate long-term deleterious cardiovascular effects.^284,285^ Intervention and therapy targeting metabolic memory and epigenetic modifications can effectively reduce the long-term damage of metabolic disorders on the circulatory system.^286^ For instance, metformin, a well-established antidiabetic drug, has been shown to effectively ameliorate the negative cardiovascular impacts of hyperglycemia by affecting the activity of numerous epigenetically modified enzymes.^287,288^

In addition to hyperglycemia, other metabolic disorders can mediate sustained cardiovascular injury through metabolic memory. Obesity could persistently affect ROS generation and cause vascular endothelial dysfunction through epigenetic modifications.^289^ Methyltransferase SUV39H1, demethylase JMJD2C, and acetyltransferase SRC-1 expression were significantly dysregulated in visceral adipose arteries of obese patients compared with normal controls.^289,290^ H3K9me2/3 and H3K9ac were correspondingly downregulated on the p66^Shc^ promoter in obese patients, increasing ROS production and decreasing NO levels. Furthermore, a hypoxic environment can trigger metabolic memory in cardiac fibroblasts, leading to cardiac tissue fibrosis.^291^ Hypoxia increases DNMT1 and DNMT3B expression by upregulating HIF-1α, which causes sustained transcriptional repression of genes, including SOD2, in fibroblasts, promoting the expression of fibrosis factors and HIF-1α.^44^ Further studies have shown that dysregulation of DNMT1 leads to normoxic HIF-1α activation by affecting SOD2.^45,292^ In addition, exposure to maternal chronic hypoxia upregulates the CpG methylation of the promoter of PKC epsilon (PKCε) in the myocardium.^293^ The expression level of PKCε is subsequently downregulated and causes increased susceptibility to ischemia and reperfusion injury in the male heart in a sex-dependent manner, which persists into adulthood.

### Endocrine system

Accumulated evidence has demonstrated the involvement of metabolic memory in the pathogenesis of various endocrine metabolic diseases. Internal and external environmental elements influence the development of endocrine tissues and organs through epigenetic alterations.^294^ These alterations can disrupt hormone secretion and action, which mediate disease progression, including metabolic syndrome, diabetes mellitus, and MAFLD.

Insulin, a crucial endocrine peptide hormone, is of great significance in modulating nutrient intake, utilization, and storage within the body. Impaired insulin secretion or reduced responsiveness of target cells to insulin serves as a causative driver of several diseases. Extensive research has shown that elevated lipid levels may contribute to the pathogenesis of insulin resistance, primarily through chronic tissue inflammation, oxidative stress, and epigenetic modifications with long-lasting effects.^295,296^ Metabolic syndrome represents a constellation of intricate metabolic disorders characterized by insulin resistance, hyperglycemia, hyperlipidemia, hypertension, and central obesity.^297^ Moreover, T2DM is closely related to impaired insulin sensitivity and insufficient insulin secretion, primarily resulting from defective pancreatic β-cell function.

Numerous epigenetic modifications have been identified in target tissues of insulin, such as skeletal muscle, adipose tissue, and liver, in case-control cohort studies on T2DM.^298–305^ These modifications include alternations in DNA methylation patterns within candidate genes correlated with T2DM like *PPARG*, transcription factor 7-like 2 (*TCF7L2*), potassium voltage-gated channel subfamily Q member 1 (*KCNQ1*), as well as insulin receptor substrate 1 (*IRS1*).^306–308^ Additionally, changes in DNA methylation were observed in obesity-induced insulin resistance compared to their non-obese counterparts. Obese individuals undergoing metabolic surgery appear to have epigenetic and metabolic changes in skeletal muscle that accompany the process of improved insulin sensitivity.^309^ You et al. identified DNMT3a as essential for mediating insulin resistance of both murine and human adipocytes.^310^ Knockout of the *DNMT3a* gene in mice adipose tissue avoids diet-associated insulin resistance yet does not result in altered adiposity.

Several investigations examined how human epigenetic modifications relate to insulin production.^263,264,311–318^ Researches unveiled the enhanced DNA methylation of *PPARGC1A*, insulin gene (*INS*), along with pancreatic and duodenum homeobox 1 (*PDX1*) in pancreatic islets of individuals suffering from T2DM in comparison to nondiabetic subjects.^312,313^ Barres et al. observed upregulated methylation and downregulation of *PPARGC1A* in skeletal muscle from T2DM patiens.^300^ PDX1 is associated with regulating early exocrine and endocrine pancreatogenesis, β cell development, as well as INS expression in mature β cells.^319,320^ Whole-genome bisulfite sequencing identified seven differentially methylated regions (DMRs) associated with T2DM islets, which supports a pivotal epigenetic regulatory role of *PDX1* transcription factor in diabetes.^264^ Mice lacking *PDX1* are prone to diabetes, while individuals mutated in *PDX1* undergo one specific diabetes type known as MODY4.^321–323^

Maternal obesity or overnutrition during pregnancy can result in epigenetic changes in organs such as the fetal hypothalamus and liver, enhancing metabolic disease risk in offspring. In addition, studies of the prolonged effects of abnormal glucocorticoid secretion have also shown that an organism’s reactivity to stress can be altered continuously through epigenetic mechanisms but that it is not possible to “turn back the clock” after the stress relieved.^324^ Early life stress results in post-stress phosphorylation of methyl-CpG-binding protein 2 (Mecp2), which diminishes the combination of MeCp2 with methylated DNA, leads to hypomethylation of arginine vasopressin (AVP) in neurons of the paraventricular nucleus of the hypothalamus and promotes *Avp* expression.^325^ Such alterations in gene expression last into adulthood, suggesting that stress from earlier life may have a persistent influence, altering how your brain responds to pressure.^326^

### Nervous system

Diabetic neuropathy is a group of clinical syndromes with diverse manifestations caused by different pathophysiologic mechanisms and widely distributed in various tissues.^327^ In general, these syndromes occurring in the peripheral nervous system can be categorized into diffuse and focal neuropathies. Diffuse neuropathies are more common and include DPN and diabetic autonomic neuropathy (DAN), which tend to be chronic and progressive. DPN is the most common type and typically presents as symmetric polyneuropathy that initially affects the distal lower extremities and progresses gradually upward as the disease progresses. Focal neuropathy is less common and is usually characterized by acute onset and self-limiting. In addition, diabetes causes degenerative changes in the central nervous system, resulting in an increased risk of cognitive impairment and Alzheimer’s disease (AD), Parkinson’s disease (PD), etc., also called diabetic encephalopathy (DE). Multiple mechanisms, including oxidative stress, chronic inflammation, and epigenetic dysregulation, are attributed to the ongoing metabolic changes in diabetic neuropathy.^327,328^

The engagement of epigenetic modifications in regulating the progression of DPN has been extensively investigated. Hyperglycemia continuously changes the state of DNA methylation and induces altered expression of genes associated with DPN. Whole genomic DNA methylation levels are dramatically reduced in leukocytes from DPN patients and may mediate insulin resistance along with low-grade inflammation to exacerbate neurologic lesions.^329–331^ Thorough analysis of methylome and transcriptome of the peroneal nerve in patients with T2D and DPN demonstrated that blood glucose levels independently and significantly affected the differential expression of the transcriptome engaged in the modulation of key aspects of DPN progression like an inflammatory response, insulin resistance, and ECM remodeling.^332,333^ For example, prostaglandin-endoperoxide synthase 1 (PTGS1), which encodes cyclooxygenase 1 (COX-1) that can promote inflammatory response and neuropathic pain, was found to be reduced methylated in patients with high HbA1c, indicating that it may function in the development of DPN.^332,334^ Increased demethylation of the promoter region of purinergic receptor P2X ligand-gated ion channel 3 (*P2X3R*) was found in dorsal root ganglion (DRG) of diabetic rats, being correlated with downregulated DNMT3a and DNMT3b.^335,336^ P2X3R can bind and activate NF-κB p65 to induce diabetic pain hypersensitivity. High glucose also induces the dysfunction of Schwann cells through DNA methylation, leading to myelin structural disorders as well as abnormalities in peripheral nerve conduction and action amplitude.^259,337^ DNMT1 and DNMT3a are upregulated in Schwann cells by the PI3K/Akt pathway suppressed by elevated glucose, thus upregulating thioredoxin-interacting protein (TXNIP) to adversely affect cellular function and metabolism.^338,339^ Treatment with DNMT inhibitor 5-Aza effectively downregulated TXNIP in the diabetic sciatic nerve, along with inhibited dysfunction and apoptosis of Schwann cells induced by hyperglycemia.

Different histone PTMs of genes associated with nerve regeneration and function are essential in DPN development. For instance, HDAC1 and HDAC5 were downregulated in rat Schwann cells stimulated by high glucose, accompanied by a decrease in autophagy markers (LC3-II/LC3-I) and abnormal myelination.^258^ Taking trichostatin A and small hairpin RNA vector as HDAC inhibitors to treat high glucose-cultured Schwann cells resulted in a significant improvement of LC3-II/LC3-I. Hyperglycemic mice showed persistent neuronal damage even after normalization of blood glucose, which could be reversed through combination therapy of glucagon-like peptide-1 receptor agonist (GLP-1RA) and metformin.^340,341^ Chen et al. revealed that GLP-1 and its analogs may attenuate FoxO-mediated oxidative stress and neurotoxicity in the hyperglycemic environment by deacetylating and phosphorylating FoxO1 through the SIRT1 and Akt pathways, respectively.^342–344^

Furthermore, ncRNAs are demonstrated to be involved in DPN progression. Various miRNAs have been identified to induce neuropathic pain and neuronal inflammation in DPN by activating the expression of pain-recognizing and pro-inflammatory-related proteins, including miR-9, miR-23, miR-146a, and miR-182, among others.^345–351^ miR-9 expression in DPN rats was reduced and accompanied by upregulation of calcium homeostasis modulator protein 1 (CALHM1), which controls extracellular Ca^2+^ influx and ATP production based on neuronal excitability.^345,352,353^ miR-9 interacts with CALHM1 to activate ATP-P2X7R signaling, which shows high expression in spinal cord and neurons, leading to neuropathic pain.^354^ In addition, DRG neuronal apoptosis in diabetic patients was related to downregulation of miR-146a.^348^ Downregulated miR-146a can upregulate the expression of IL-1 receptor-activated kinase (IRAK1) and tumor necrosis factor receptor-associated factor-6 (TRAF6) to mediate NF-κB overactivation, upregulating pro-inflammatory factors such as TNF-α and IL-1β along with DPN progression.^349^ Several recent studies have highlighted specific lncRNAs (NONRATT021972, uc.48+, and BC168687) as key factors in the pathogenesis of diabetic neuropathy and neuropathic pain.^355–357^ Targeting these particular lncRNAs in diabetic rat models attenuates DPN and may help ameliorate neuronal impairment potentially through modulation of inflammatory factor production.^358–361^

Diabetic encephalopathy is one complication of diabetes related to the central nervous system, usually associated with neurodegenerative lesions and cognitive impairment.^362,363^ The interaction between diabetes and cognition is complicated by the influence of epigenetic factors.^364,365^ On the one hand, persistent insulin resistance in peripheral tissues of metabolic syndrome leads to dysregulation of insulin receptor pathway activity in the brain, which ultimately results in brain insulin resistance.^366^ Brain insulin resistance increases the accumulation of Aβ and tau hyperphosphorylation, causing intense inflammation and neurodegeneration of nerve cells, which is considered to be an important pathogenetic component of AD.^341,366,367^ Histone modifications have been shown to intervene in the progression of AD by modulating autophagy in neuronal cells. On the other hand, an aberrant metabolic environment itself leads to changes in epigenetic regulation, which mediates the continued progression of metabolism-related neurodegenerative diseases. HDAC4 is linked to the pathologic process of several neurodegenerative ailments, and its expression is elevated in high glucose-treated hippocampal neurons.^368^ Radix polygoni multiflori downregulates HDAC4 in diabetic encephalopathic rats and reduces the apoptosis of hippocampal neurons by suppressing the activity of c-Jun aminoterminal kinase (JNK) pathway, which ultimately improves the cognitive function.^369^ In addition, miRNAs play important regulatory roles in metabolic memory-mediated neurodegenerative diseases.^370–373^ For example, miR-132 is downregulated in hippocampal neurons in rats with diabetic encephalopathy, accompanied by upregulated glycogen synthase kinase (GSK)-3β and tau. miR-132 could alleviate the impairment of diabetic encephalopathy via inhibiting GSK-3β expression and attenuating Tau hyperphosphorylation.^374^ A recent study also revealed significant alterations of multiple circRNAs in hippocampal neurons of diabetic mice.^375^ circ-Nbea is significantly downregulated by hyperglycemia and may facilitate the progression of DE by sponging miR-128-3p.^376,377^

### Urogenital system

Diabetic kidney disease (DKD) is one high-risk complication of diabetes.^378^ Several experimental and clinical research revealed the association between CpG DNA methylation and DKD.^379^ Differential methylation of several genes, including *UNC13B*, has been identified in diabetic individuals with DKD in comparison to those without DKD, with *UNC13B* being associated with hyperglycemia-induced apoptosis in glomerular cells.^116^ The persistence of differential methylation of *TXNIP* loci was detected in whole blood and monocyte samples from the same DCCT/EDIC patients collected 16–17 years apart, which is thought to deliver glucose stress to promote oxidative stress and apoptosis, playing a pivotal role in DKD progression.^113,380,381^ Upregulation of TGF-β1 enhances Ras activation in fibroblasts by inducing hypermethylation of RAS protein activator like-1 (*Rasal1*) promoter, which promotes cell proliferation and fibrosis.^382^ TET3-mediated hydroxymethylation reverses the changes in *Rsasl1* promoter DNA methylation pattern to reduce fibrosis.^383^

Studies investigating histone PTMs at critical metabolic genes suggested the critical role of epigenetic regulation in DKD pathogenesis. Histone modifications engage in DKD progression by affecting aspects of fibrosis, oxidative stress, and inflammation. For example, histone methylation at different locations in diabetic glomerular thylakoid cells is altered, exhibiting an increase in H3K4me1/2/3, H3K36me2/3, and H3K79me2 which promotes inflammatory responses and ECM accumulation, while accompanied by a decrease in H3K9me2/3 and H3K27me3 which are involved in the inhibition of renal fibrosis process.^141,384,385^ In addition, renal inflammation-mediated DKD progression is associated with histone modifications, which are involved in activating inflammatory factors like NF-κB by macrophages and monocyte infiltration under hyperglycemia.^386^ Epigenome profiling investigations revealed variations in H3K4me2 and H3K9me2 for genes relevant to diabetes and inflammation in monocytes exposed to hyperglycemia versus normoglycemic controls.^387,388^ Similar epigenetic changes were found in blood monocytes and lymphocytes of T1DM patients. HMT SET7 has been identified as a promoter of H3K4 methylation, which could co-activate pro-inflammatory genes downstream of NF-κB in monocytes.^389^ The association between histone acetylation modifications and DKD has been increasingly studied.^390^ p300, CBP and other HATs expression is upregulated in DKD and mediates increased transcription of pro-inflammatory and pro-fibrotic factors that exacerbate glomerular dysfunction associated with DKD.^391–393^ Inhibition of p300/CBP effectively reduces H3K9/14Ac levels, offering a potential therapeutic approach for managing hyperglycemia-mediated long-term kidney injury.^394^

ncRNAs also serve as essential regulators in the progression of DKD. miR-192 was first found to be downregulated in DKD, which targets TGF-β/Smad3 to upregulate ECM and collagen, as well as to promote renal fibrosis.^395–399^ Several miRNAs are believed to modulate vital features of DKD, such as apoptosis, fibrosis, and hypertrophy.^244^ miR21, miR-34a-5p, miR217, and others were found to be elevated under high glucose induction, combined with the promotion of inflammatory pathway signaling and induction of oxidative stress along with tissue fibrosis.^400–402^ Studies in recent years have also implicated lncRNAs in DKD.^403^ LncRNA plasmacytoma variant translocation 1 (*PVT1*) has been found to contribute to fibrosis and DKD pathogenesis.^404^ Upregulated PVT1 of glomerular thylakoid cells is correlated with increased cellular expression of pro-fibrotic elements under hpyerglycemia.^404–406^

Furthermore, as previously mentioned, metabolic disorders such as dietary factors have been implicated in the reduction of sperm quality in men and would be inherited intergenerationally through epigenetic modifications.^37^ High-fat diet during early paternal years induces permanent alterations in testicular lipid content and leads to irreversible sperm quality damage, which can persist for up to two generations.^38,407^

### Immune system

Cancer is intimately tied to dysregulated immune homeostasis. There is substantial evidence indicating that metabolic disorders such as obesity and diabetes are correlated to an increased susceptibility to a number of cancers, including endometrial, liver, pancreatic, colorectal, and breast cancers.^408^ These metabolic abnormalities associated with obesity and diabetes have been widely recognized as risk factors for cancer morbidity and mortality. Further studies have demonstrated that cancer cell proliferation, migration, invasion, and resistance to chemotherapy are enhanced in a hyperglycemic environment, and this enhancement persists after blood glucose level is normalized.^409^ This phenomenon suggests that sustained regulation of metabolic memory has an essential impact on metabolic diseases and cancer.^410^

Recent studies have pointed out the occurrence of diverse epigenetic modifications of cancer cells under an environment of metabolic abnormalities. For instance, in breast cancer cell line MDA-MB-231, exposure to a hyperglycemic environment triggers dephosphorylation and activation of GSK-3β, which is vital in mediating insulin-dependent glycogen synthesis.^411^ Upregulation of GSK-3β enhances phosphorylated imprinting of histone H3, which mediates the upregulation of metastatic genes along with promoting epithelial-mesenchymal transition (EMT) and cancer cell proliferation. Additional studies have revealed that high glucose mediates overexpressed neuromodulin 1 (Nrg1) in breast cancer, fueling malignant cancer growth via the epidermal growth factor receptor (EGFR) pathway.^412^ Subsequent investigations have identified increased active enhancer modifications, including H3K4me1 and H3K27ac, in Nrg1 enhancers after hyperglycemia treatment and were consistent with the upregulation of Nrg1 expression.^413^ These findings indicate the persistence of this epigenetic imprint even after cancer cells return to normoglycemic conditions, facilitating aggressive tumor growth.^414^

A recent study has revealed an intriguing discovery that drug-resistant residual cells in microscopic residual disease of breast cancer exhibit similar DNA methylation status as cancer cells, regardless of lacking proliferative phenotypes and oncogenic signals.^415^ Notably, the promoter regions of HIF-1α, which is commonly recognized as an active master modulator of tumor glycolysis, displayed a comparable DNA methylation profile. Furthermore, both glycolytic co-activator proteins and glycolytic target genes of HIF-1α exhibited consistent demethylation patterns in cancer as well as residual cells, and isozyme-specific demethylation was present in either type of cells. These findings suggest that even after the removal of metabolically aberrant oncogenic signals from the environment, residual cells retain cancer-associated metabolic traits through epigenetic modifications and favor cancer resistance and recurrence.

In addition, metabolic memory can modulate cancer development through the influence of AGEs, oxidative stress, and inflammation. Pan et al. demonstrated that in diabetic patients, glycosylation and oxidation for high-density lipoprotein (HDL) may contribute to abnormal enhanced adhesion of breast cancer cells to human umbilical vein endothelial cells or ECM, thereby facilitating breast cancer metastasis.^416^ Moreover, Nε-(1-carboxymethyl)-L-lysine (CML), an AGE, was found to enhance the formation of chondrosarcoma tumor spheroids and upregulate cancer stem cell activity.^417^ CML treatment also augmented the migratory and invasive capability of tumor cells by advancing the EMT process. These observations suggest a potential promotional effect of AGEs on the stemness and metastatic properties of cancer.

The discovery of metabolic memory mechanisms opens up new possibilities to explain the higher risk and difficulty in treating cancers coupled with metabolic disorders. Targeting metabolic memory to develop assays and therapeutic agents in conjunction with conventional anti-tumor therapies could be a promising cancer therapeutic strategy for the future.

## Prospects for metabolic memory applications

Given that the molecular and cellular mechanisms of metabolic memory mediate the long-term adverse effects of metabolic disorders, simply controlling the initial metabolic disorders is clearly insufficient. The development of disease diagnostic and therapeutic measures based on the molecular mechanisms of metabolic memory, especially epigenetic modulation, is a highly promising direction. Here, we describe current research progress in applying metabolic memory for disease diagnosis, biomarker identification, treatment, prevention, and prognosis. In addition, we also summarize clinical trials in metabolic diseases that have investigated metabolic memory-related mechanisms for screening, treatment, and prevention of diseases and complications in Table 1.

**Table 1:** Clinical trials targeting metabolic memory-related mechanisms in metabolic diseases

Study Type	Primary Purpose	Disease or Problem	Intervention or Exposure	Possible Mechanism or Target	Primary Outcome	Actual Enrollment	Result	Clinical trial number or reference
*Interventional*	Treatment	Type 1 Diabetes Mellitus	Behavioral: Insulin	Advanced glycation end/products	The appearance and progression of retinopathy and other complications	1441	In the DCCT/EDIC, the benefit of early intensive therapy intervention in young adults with recent/onset type 1 diabetes, and the observed delayed effect of intensive glycemic control on diabetic complications by influencing DNA methylation exemplify the metabolic memory phenomenon	NCT00360815^111,501–505^
		Type 1 Diabetes Mellitus	NO	Epigenetic Regulation	The risk factors for Micro/ and cardio/vascular complications including genetic and epigenetic factors	1441	Epigenetic DNA methylation (DNAme) has been shown to be involved in metabolic memory	NCT00360893^111^
		Type 2 Diabetes Mellitus	Behavioral: Aerobic Training, Resistance Training	Advanced glycation end/products	Change in hemoglobin A1c (HbA1c) levels [Time Frame: 4 months]	40	Exercise training in T2DM patients was shown to affect the expression of c/miRNAs and regulate metabolism	NCT01182948^460^
		Type 2 Diabetes Mellitus	Drug: RVX000222	Metabolic control	The time from randomization to the first occurrence of adjudication/confirmed major adverse cardiovascular events (MACE)	2400	Apatadione/mediated BET proteins reduce CNS disease risk, but not cardiovascular disease risk significantly, by recognizing histone acetylation residues	NCT02586155^506–508^
		Type 2 Diabetes Mellitus	Behavioral: Stop smoking, Diet, Exercise Drug: Blood pressure lowering therapy, Glucose lowering therapy, Lipid lowering therapy	Metabolic control	Years of life years gained [Time Frame: 21 years] Diabetic nephropathy [Time Frame: 4 years] Combined cardiovascular endpoint [Time Frame: 8 years] Total mortality [Time Frame: 13 years]	160	The intensive intervention had beneficial effects on cardiovascular causes	NCT00320008^34^
		Type 2 Diabetes Mellitus, Other Disorders of Bone, Density and Structure	Dietary: Resveratrol	Epigenetic Regulation	C reactive protein (CRP) [Time Frame: up to 25 months]	192	Sirtuin1 (SIRT1) reduces the epigenetic marker H3K56 by histone deacetylase	NCT02244879^509^
		Breast Cancer, Epigenetic Disorder, Obesity	Behavioral: Group educational intervention program (IGOBE)	Epigenetic Regulation	DNA methylation levels, Concentration of inflammatory biomarkers, Concentration of Oxidative stress biomarkers, etc. [Time Frame: 4 months]	220	No study results posted	NCT01248286
		Diabetic Nephropathy	Drug: Calcium dobesilate	Epigenetic Regulation	Urinary albumin creatinine ratio, Changes in miRNA expression	160	No study results posted	ChiCTR/IPR/17013639
		Type 2 Diabetes Mellitus	Drug: Huangqi simiao decoction	Epigenetic Regulation	Traditional Chinese Medicine Syndrome Score	60	No study results posted	ChiCTR2300077151
	Screening	Type 1 Diabetes Mellitus	Genetic: Detection of RAGE gene polymorphism (rs1800625)	Advanced glycation end/products	Correlation of RAGE gene polymorphism rs1800625 with the incidence of type I diabetes [Time Frame: 4 months]	354	No study results posted	NCT05874323
	Prevention	Cardiovascular disease, Circulatory System, Cardiovascular disease	Dietary: Virgin olive oil/ Nuts/ Low/fat diet	Epigenetic Regulation	A composite endpoint of cardiovascular death, non/fatal myocardial infarction, and non/fatal stroke.	7500	Mediterranean diet regulates lipid metabolism by influencing DNA methylation	ISRCTN35739639^510,511^
		Obesity, Cardiovascular Diseases, Heart Diseases	Behavioral: Four Diets Differing in Macronutrient Composition, Diets Low in Saturated Fat	Epigenetic Regulation	Change in body weight (measured at Year 2)	811	Dietary fat intake influences epigenetic inheritance of the DNA Methylation of NFATC2IP	NCT00072995^512^
*Observational*	Screening	Type 2 Diabetes Mellitus, Diabetic Kidney Disease	NO	Epigenetic Regulation	Urine and serum expression of miR/192 and miR/25[Time Frame: Each patient will be assessed at baseline.]	300	No study results posted	NCT04176276
		Gestational Diabetes	Other: blood draw	Advanced glycation end/products	miRNA profile [Time Frame: within 1 month after blood drawn]	160	No study results posted	NCT05632055
		Gestational Diabetes Mellitus, Macrosomia	NO	Epigenetic Regulation	Fetal macrosomia was identified, DNA methylation level in macrosomia [Time Frame: 40 weeks], GDM was identified [Time Frame: 24/28 weeks]	239	No study results posted	NCT03165643
		Ischemic stroke	NO	Epigenetic Regulation	EMV	100	No study results posted	NCT03905434
		miRNA	NO	Epigenetic Regulation	Composite cardiovascular outcome [Time Frame: up to 10 years]	460	No study results posted	NCT03635255
		Type 1 Diabetes Mellitus	NO	Epigenetic Regulation	Diabetes/prediabetes, Cognitive functions, Overweight, etc. [Time Frame: 0 / 18 years]	584	No study results posted	NCT01559181
		Type 1 Diabetes Mellitus, Type 2 Diabetes Mellitus,	NO	Epigenetic Regulation	Body mass density, HR/pQCT and DXA	80	No study results posted	DRKS00022762
		Type 2 Diabetes Mellitus	NO	Epigenetic Regulation	Exosome proteome signatures [Time Frame: 3 years]	100	No study results posted	CTRI/2020/12/029975
		Type 2 Diabetes Mellitus	NO	Epigenetic Regulation	DNA methylation [Time Frame: 1 hr]	158	No study results posted	NCT02316522
		Type 2 Diabetes Mellitus	NO	Epigenetic Regulation	DNA methylation and gene expression [Time Frame: 1 h]	249	No study results posted	NCT02021695
		Type 2 Diabetes Mellitus, Cancer, Hepatocellular Carcinoma	NO	Epigenetic Regulation	LncRNA H19 & IGF/1 R mRNA expression [Time Frame: baseline]	101	No study results posted	NCT04767750
		Diabetic retinopathy (DR)	NO	Epigenetic Regulation	ANRIL, Ang, AT1R, etc.	100	lncRNA/ANRIL can predict the development of DR	ChiCTR1800017500^513^
		Cervical cancer	NO	Epigenetic Regulation	Cervical Cancer	183	No study results posted	ChiCTR2000034241
	Prevention	End/stage Renal Disease, Chronic Kidney Disease	NO	Epigenetic Regulation	Adverse cardiovascular events [Time Frame: 3 years]	360	No study results posted	NCT02304471

### Disease biomarkers

Due to the presence of metabolic memory, the onset and progression of complications may occur when early symptoms of metabolic disorders are not apparent in the patient and may persist after the metabolic disorder has been controlled.^418,419^ Early diagnosis and treatment of metabolic disorders is critical to the management and prognosis of metabolic diseases. Mounting evidence suggests that various epigenetic modifications are intimately associated with disease development as primary molecular mechanisms of metabolic memory.^286,420^ Epigenetic changes have been recognized to hold promise as sensitive, reliable, and easily accessible biomarkers to predict the risk of disease progression, playing a crucial role in early diagnosis and prognosis of metabolic disorders.

Research on DNA methylation biomarkers in metabolic memory-related diseases has flourished with considerable progress. For example, with the progress of a high-throughput epigenome-wide association study (EWAS), the results of differentially methylated genes were identified by microarray chip methylation analysis and microbead array chip methylation analysis revealed differentially methylated sites related to the risk of developing diabetes, and its complications, including *TCF7L2*, *KCNQ1*, *TXNIP*, ATP-binding cassette subfamily G (*ABCG1*), phosphoethanolamine/phosphocholine phosphatase (*PHOSPHO1*) and others.^259,421–426^ It has been shown that DNA methylation status in peripheral blood can be detected noninvasively from cells by methylation-specific PCR and is a promising marker for the detection of metabolic disorder-related diseases.^427,428^ In addition, DNA methylation modifications have been shown to correlate with β-cell dysfunction or death.^429^ The *INS* gene, which encodes the insulin precursor, is expressed chiefly in the β-cells of the pancreatic islets and exhibits unmethylation, whereas it is methylated in other tissues.^430^ When β-cells are disrupted by metabolic diseases, unmethylated *INS* DNA is shed into the circulation and can be detected by a variety of PCR-based molecular methods.^431–435^ Individuals with recent-onset type 1 diabetes (T1D) and those at-risk groups demonstrated notably elevated levels of unmethylated INS DNA in comparison to nondiabetic controls.^436^ Detection of unmethylated *INS* DNA by blood samples can be effective for early diagnosis of T1D and screening of high-risk subjects.^437^ Unmethylated INS DNA can also be used to monitor rejection and prognosis of islet transplantation. Immediate elevation of *INS* DNA levels occurs after islet transplantation and its persistent elevation may indicate a greater likelihood of long-term functional loss of the graft.^438^ Several epigenomic association studies of hepatic fat have shown that DNA methylation changes of peripheral blood origin are strongly associated with hepatic fat accumulation and could be attractive biomarkers for the detection of MAFLD.^439,440^

In addition, alternations in the levels of numerous miRNAs and lncRNAs are correlated with disease progression, being potential targets for disease diagnosis as well.^98,441,442^ For instance, examination of plasma circulating miRNA profiles, particularly changes in plasma levels of miR-150, miR-30a-5p, miR-15a, and miR-375, in the years prior to the onset of T2DM and prodromal DM can be utilized to assess the risk of developing T2DM.^443^ This may improve prediction and prevention in people at risk for T2DM. In addition, recent findings have identified a series of lncRNAs that are dysregulated in the adipocytes of obese mice fed with a high-fat diet, which showed dynamic alternations in the fed versus fasted state.^444^ Among them, lnc-ORIA9 (lnc-leptin) is strongly associated with of leptin, which is essential for adipogenesis and can potentially serve as a novel molecular indicator of adipose energy status.

### Therapeutic targets

#### Behavioral interventions

Various lifestyle or environmental exposures (including malnutrition, high-fat diets, and physical inactivity) are important factors influencing the formation of metabolic memory.^445^ Lifestyle interventions such as appropriate dietary modifications and exercise are known to have important health benefits for metabolic homeostasis and the immune system and may be beneficial for preventing or mitigating the risk of metabolic diseases.^446^ Recent studies have identified ways in which dietary modifications can intervene in the possible long-term adverse effects mediated by metabolic memory by influencing epigenetic modifications.^447,448^ Dietary bioactive compounds such as polyphenols and terpenoids serve as epigenetic modifiers, and appropriately increasing intake of them may reverse epigenetic alterations in metabolism-related genes in offspring from a poor maternal diet.^449,450^ Specifics on increasing the intake of dietary bioactive compounds for the intervention of metabolic memory-related disorders will be presented in the next section.

A sedentary lifestyle or lack of exercise is one of the main risk factors for the progression of diseases related to metabolic disorders.^451^ Past studies have shown that exercise is beneficial in ameliorating insulin resistance, which has recently been shown to be possibly correlated with altered epigenetic modifications.^452^ It was shown that promoter methylation levels of genes, including peroxisome proliferator-activated receptor gamma coactivator 1 (*PGC1*), *MEF2*, and *PPARγ*, are reduced immediately after acute exercise and lead to a subsequent increase in the expression of their genes.^453^ PGC1 is decreased in insulin-resistant skeletal muscle, which induces mitochondrial dysfunction, leading to increased intracellular lipid levels in myocytes and further insulin resistance.^454^ Exercise may improve the persistence and progression of insulin resistance by upregulating PGC1 expression and activity in skeletal muscle. A study analyzing DNA methylation changes in adipose tissue of healthy subjects before and after a six-month exercise intervention showed significant alterations in CpG methylation levels of several obesity- and diabetes-related genes, accompanied by simultaneous changes in mRNA expression.^455^ Sedentary patients with metabolic diseases also showed hypomethylation of the promoter regions of genes such as NRF1, solute carrier family 27 member 4 (SLC27A4), 6-phosphofructo-2-kinase/fructose-2,6-bisphosphatase 3 (PFKFB3), and GSK3A in skeletal muscle after 16 weeks of aerobic training, which is thought to contribute to the reduction of circulating lipids and improved glucose metabolism.^456^ In addition, multiple types of physical exercise modulate miRNA expression in healthy and metabolic disease patients.^457–459^ Aerobic or resistance exercise training was associated with substantial circulating mi-RNA alterations in diabetic patients and significantly upregulated miR-423-3p, miR-451a, and miR-766-3p. miR-451a and miR-423-3p target fatty acid biosynthesis and metabolism regulation and were significantly associated with fat loss. Physical exercise also reversed some miRNAs that are aberrantly expressed in T2DM.^460^ Chronic physical exercise downregulated miR-144, which is overexpressed in diabetes, and ameliorated miR-144-mediated insulin resistance through altered IRS1 and IRS2 expression.^461–463^ In contrast, downregulation of miR-15a and miR-192 in the plasma of T2DM patients promoted insulin resistance and disease progression.^443,464,465^ Physical exercise leads to increased expression of miR-15a and miR-192 to ameliorate insulin resistance and lipid accumulation.^466,467^

#### Natural products

Despite the alarming long-term effects of metabolic disorders and their complications, advances in research on natural products of foods and drugs offer new insights on the treatment of metabolic diseases.

The monoterpenes, sesquiterpenes, phenolics, and diarylheptaterpenes (curcumin) extracted from ginger, widely used as food and medicine in daily life, are involved in the regulation of apoptosis, cell cycle/DNA damage, chromatin/epigenetic regulation, cytoskeletal regulation, and adhesion through specific signaling pathways, which are beneficial for diabetes and metabolic syndrome, cholesterol levels and lipid metabolism, and inflammation.^468^ Curcumin is a natural product and a p300/CBP-specific HAT inhibitor involved in suppressing cellular histone acetylation levels.^469^ In endothelial cells, curcumin has shown the ability to prevent hyperglycemia-induced histone acetylation in the promoter regions with important regulatory roles in vascular structure and function under diabetic conditions.^470^ Garcinol, which can also act as a HAT inhibitor, reduces the transcription of inflammatory factors by inhibiting the hyperglycemia-induced enhancement of histone H3 acetylation.^471^

Astragalus is also considered a promising drug containing prominent bioactive natural saponins, such as Astragaloside IV (AST) and cyclosiversioside F (CSF). AST decreased RSC96 cell apoptosis and alleviated DPN in rats through the miR-155-mediated PI3K/Akt/mTOR signaling pathway.^472^ CSF attenuates insulin resistance, adipocyte inflammation, and apoptosis through PI3K/Akt, NF-κB, and MAPK signaling.^473^

What’s more, long-term dysfunction of pancreatic β-cells induces insulin resistance and chronic complications. Genistein with structural specificity directly regulates β-cell proliferation and insulin secretion and protects against apoptosis through the cAMP/PKA pathway.^474,475^ Polyphenolic compounds in fruits and grains protect β-cells under high glucose conditions through the inhibition of histone acetylation.^476–480^ Furthermore, resveratrol and sirtuins treat metabolic disorders in epigenetic regulation of DNMT, HDAC, and lysine-specific demethylase-1 (LSD1).^481,482^ Resveratrol can reverse hyperglycemia-induced AMPK inactivation by activating HDAC SIRT1, inhibiting the NF-κB pathway and pro-inflammatory factors.^483^ Glucose metabolism and insulin sensitivity were significantly improved in T2DM patients receiving resveratrol treatment.^484^

Epigenetic modulation of cell function and metabolism by various natural products provides a new therapeutic approach to eliminating the long-term effects of metabolic disorders. The effect of the dose of various natural ingredients on metabolic memory, as well as synergistic or antagonistic effects between components, warrant further study.

#### Targeted drugs

A number of novel drugs that target epigenetic changes to eliminate the long-term effects of metabolic memory are being developed.^485^ These drugs are named epigenetic drugs (or epidrugs) and mainly include HAT inhibitors (HATi), HDAC inhibitors (HDACi), HDAC activators, and miRNA inhibitors.^486,487^ Epigenetic drugs have not been formally approved for metabolic disease treatment, but there have been several epigenetic drugs approved for use in other diseases related to metabolic diseases, including decitabine, valproic acid (VPA), vorinostat, and so on.^488–490^ It is reasonable to believe in the promising efficacy of drugs targeting epigenetic regulation for metabolic diseases and metabolic memory, but there is still a need to address the systemic and low specificity of current epigenetic drug targets to make epigenetic therapies fittingly appropriate for metabolic memory.^491–493^ Targeting miRNAs for inhibition is also considered a promising therapy, such as locked nucleic acid (LNA) modification. In diabetic mice, the specific inhibition of miR-192 using LNA-anti miR-192 demonstrated a reduction in the expression of pro-fibrotic genes, leading to the reversal of persistent renal fibrosis.^396^ In addition, miRNA delivery via extracellular vesicles (EVs) has been shown to be effective in preventing the progression of metabolic complications in animal models.^494^ Adipose-derived stem cells (ADSC) secreted EVs with high expression of miR-130a-3p compared with DPN patients and rats. Treatment with ADSC-derived EVs in DPN rats resulted in a targeted decrease of DNMT1 expression and activation of the NRF2/HIF-1α/skeletal muscle actin alpha 1 (ACTA1) pathway, which ultimately contributed to the repair of peripheral vascular injury.^495^ miR-20b-3p is specifically enriched in plasma-derived exosomes of healthy rats (hplasma-exos).^495,496^ The injection of hplasma-exos into rats with diabetic peripheral neuropathy (DPN) resulted in an upregulation of miR-20b-3p levels in the sciatic nerve, in turn, mitigated autophagic damage induced by high glucose in Schwann cells and improved DPN symptoms.^497^

The development of novel drugs targeting metabolism-related epigenetic changes in cancer and combining them with conventional cancer therapies will help to inhibit cancer proliferation, metastasis, and drug resistance. As mentioned earlier, metabolic memory ties metabolism-related diseases to cancer, and the development of drugs targeting epigenetic modulators has gathered significant interest in the field of cancer research.^498^ Restoring chromatin structure to its original state through epigenetic therapies is a major research focus in cancer treatment. Epigenetic therapies hold promise in reversing abnormal imprinting events in chromatin and may provide a novel approach to treating the memory effects of hyperglycemia. HMT inhibitors are reported as a key candidate for cancer therapies by recent preclinical studies.^499^ The administration of the lysine inhibitors tazemetostat, SHR2554, and pinometostat exhibited satisfactory therapeutic efficacy in several cancers with an acceptable safety profile.^500^ Further studies are still required to explore possible avenues for targeting metabolic memory in combination with cancer treatment.

## Conclusion

“Metabolic memory” is originally defined as the long-term adverse effects of hyperglycemia even after glycemic normalization. In this review, we outline the research history and put novel insights to the salient features of metabolic memory from molecular cellular aspects to different organs and systems levels. The early metabolic abnormalities leave memory imprints on target cells, which persistently affect cellular function at multiple levels and finally induce multisystem diseases. The persistent harmful effects include inflammatory changes, premature cell senescence, and apoptosis. The enduring adverse effects eventuate metabolic diseases on different tissues and organs. Early intensive metabolic control has long-lasting benefits in reducing the risk of these complications, even after patients return to standard metabolic control. we further summarize recent advances in metabolic memory phenomena and their associated molecular mechanisms as indicators for detecting metabolic diseases and targets for therapeutic interventions. This phenomenon probably implies the potential reasons for metabolic diseases and their chronic progressive complications.

In addition, we emphasize the presence and impact of metabolic memory in a variety of metabolic diseases and establish a link between molecular mechanisms and disease progression. Metabolic reprogramming and epigenetic modifications are critically involved in the of biological mechanism of metabolic memory. They both interact and work together. Long-lasting epigenetic changes induced by metabolic stimuli could be the pivotal mechanism underlying metabolic memory. Epigenetic modifications could induce persistent expression of metabolic disease-related genes and pro-inflammatory genes, while the accumulation of some products may be the source of epigenetic changes. DNA methylation, as well as histone modification and non-coding RNA, have been implicated in the progression of metabolic complications. Early metabolic control can prevent the development of epigenetic changes that contribute to the alleviation of excessive oxidative stress and inflammation. The evaluation of epigenetic changes and metabolic reprogramming will lead to the discovery of the pathogenesis of metabolic diseases and complications.

Although the concept of metabolic memory initiates from the progression of diabetic complications, it is nowadays extended to the legacy effects of not only hyperglycemia but also hyperlipidemia and other detrimental metabolic environments. The existence of the metabolic memory phenomenon has challenged conventional therapeutic approaches to metabolic diseases. Based on the importance of early intervention in metabolic disease management, metabolic memory phenomena, and their associated molecular mechanisms might indicate new ways and targets for therapeutic interventions. Variations in circulating DNA methylation levels may be a potential tool to aid in the early screening and prognostic assessment of metabolic diseases. Targeting epigenetic modifications for behavioral interventions, dietary supplementation, and pharmacotherapy are promising future directions for investigation and development. At the same time, there are still confusions that need to be clarified in the field of metabolic memory. For example, more analysis is needed to precisely detect the persistent time and severity in different metabolic abnormalities. It is also of great significance to investigate the potential differences in metabolic memory according to age variations. For patients with metabolic syndrome comorbid with AD, cancer, or other age-related diseases, it is necessary to develop combined therapies targeting epigenetic changes to help these patients achieve better outcomes and prognosis. Also, more clinical trials and biological evidence are needed to explore the mechanisms of metabolic memory and its association with diseases in the future.
